# Rational design of a SOCS1-edited tumor-infiltrating lymphocyte therapy using CRISPR/Cas9 screens

**DOI:** 10.1172/JCI163096

**Published:** 2023-12-15

**Authors:** Michael R. Schlabach, Sharon Lin, Zachary R. Collester, Christopher Wrocklage, Sol Shenker, Conor Calnan, Tianlei Xu, Hugh S. Gannon, Leila J. Williams, Frank Thompson, Paul R. Dunbar, Robert A. LaMothe, Tracy E. Garrett, Nicholas Colletti, Anja F. Hohmann, Noah J. Tubo, Caroline P. Bullock, Isabelle Le Mercier, Katri Sofjan, Jason J. Merkin, Sean Keegan, Gregory V. Kryukov, Caroline Dugopolski, Frank Stegmeier, Karrie Wong, Fiona A. Sharp, Louise Cadzow, Micah J. Benson

**Affiliations:** KSQ Therapeutics, Lexington, Massachusetts, USA.

**Keywords:** Immunology, Therapeutics, Cancer immunotherapy, Gene therapy, T cells

## Abstract

Cell therapies such as tumor-infiltrating lymphocyte (TIL) therapy have shown promise in the treatment of patients with refractory solid tumors, with improvement in response rates and durability of responses nevertheless sought. To identify targets capable of enhancing the antitumor activity of T cell therapies, large-scale in vitro and in vivo clustered regularly interspaced short palindromic repeats (CRISPR)/Cas9 screens were performed, with the SOCS1 gene identified as a top T cell–enhancing target. In murine CD8^+^ T cell–therapy models, SOCS1 served as a critical checkpoint in restraining the accumulation of central memory T cells in lymphoid organs as well as intermediate (Tex^int^) and effector (Tex^eff^) exhausted T cell subsets derived from progenitor exhausted T cells (Tex^prog^) in tumors. A comprehensive CRISPR tiling screen of the *SOCS1*-coding region identified sgRNAs targeting the SH2 domain of SOCS1 as the most potent, with an sgRNA with minimal off-target cut sites used to manufacture KSQ-001, an engineered TIL therapy with SOCS1 inactivated by CRISPR/Cas9. KSQ-001 possessed increased responsiveness to cytokine signals and enhanced in vivo antitumor function in mouse models. These data demonstrate the use of CRISPR/Cas9 screens in the rational design of T cell therapies.

## Graphical abstract

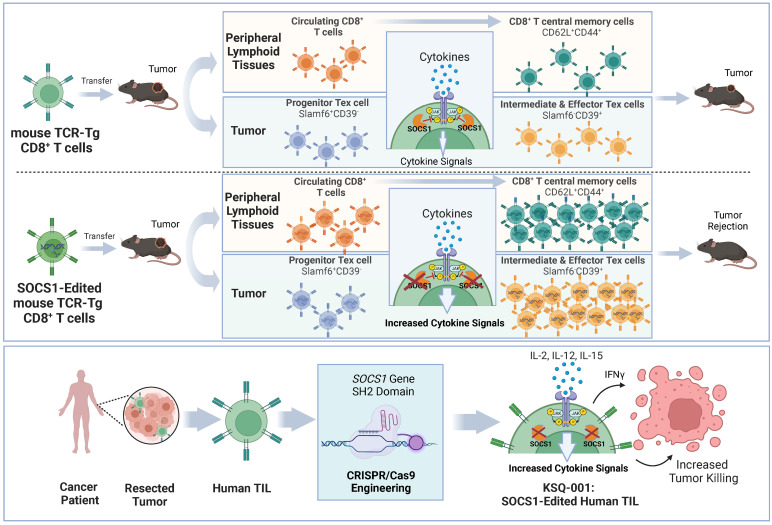

## Introduction

Cell therapies using T lymphocytes possessing antitumor specificity have demonstrated robust clinical activity in hematological malignancies (1–4), with more limited success observed in solid tumors (5–7). The reduced effectiveness of T cell therapies in solid tumors is thought to be due, in part, to the immunosuppressive nature of solid tumor and its blunting impact on the function of transferred T cells. Despite this challenge of tumor-driven immunosuppression, T cell therapies using tumor-infiltrating lymphocytes (TILs) derived from a patient’s tumor have demonstrated promise in certain clinical settings, such as in refractory metastatic melanoma, with objective responses observed in patients who have failed multiple rounds of therapy, including with immune-checkpoint inhibitors (8–11). However, the majority of melanoma patients receiving TIL ultimately progress, and fewer objective responses have been observed in other solid tumors, such as in cervical (12), ovarian (13), and non–small cell lung cancer (NSCLC) (14), where the immunosuppressive tumor microenvironment (TME) barrier may be more stringent then in melanoma. Strategies are thus sought to enhance the antitumor potency, accumulation, persistence, and memory formation of TIL and other T cell therapies, as enhancement in these functional attributes are predicted to directly translate to increased durable objective responses. It is currently unclear which T cell pathways are most impactful for targeting in enhancing the antitumor function of T cell therapies against solid tumors.

Functional screens constitute a powerful approach to discovering novel biology in disease-relevant mammalian models. The ability of the clustered regularly interspaced short palindromic repeats (CRISPR)/Cas9 gene-editing system to precisely inactivate a target gene at the level of genomic DNA has enabled the large-scale interrogation of gene function by use of pooled loss-of-function screens (15–17). This approach has been notably used for the discovery of genetic vulnerabilities and therapeutic targets in cancer types (18) and in immune cell types in various settings, including the mapping of innate cells in response to proinflammatory stimuli (19, 20), pathways involved in regulating T cell function (21–24), and Treg function (25, 26). CRISPR/Cas9 functional genomic screens thus afford the opportunity to comprehensively map the biology of primary T cells by querying gene targets driving predefined functions.

In this study, we used large-scale CRISPR/Cas9 screens to identify top T cell therapy–enhancing targets, with SOCS1 identified as a top hit. SOCS1 serves as a negative regulator of cytokine signaling in T cells, including IL-2, IL-12 and IL-15, with these cytokines known to influence the survival, differentiation, and function of T cells (27, 28). SOCS1 was found to be a checkpoint in transferred CD8^+^ T cells restraining the accumulation of CD44^+^CD62L^+^ T central memory (Tcm) cells within peripheral lymphoid organs as well as in the differentiation of Slamf6^+^CD39^–^PD-1^med^ progenitor T exhausted (Tex^prog^) cells into Slamf6^–^CD39^+^PD-1^hi^ intermediate (Tex^int^) and effector (Tex^eff^) subsets within tumors. Based on these insights, we used a SOCS1 protein domain CRISPR/Cas9 tiling screen to develop KSQ-001, an engineered TIL (eTIL therapy with inactivation of the *SOCS1* gene via CRISPR/Cas9 editing that demonstrated enhanced production of IFN-γ, increased responsiveness to cytokine signals, and increased antitumor activity in a solid-tumor TIL model). The use of CRISPR/Cas9 functional genomic screens in the rational design of engineered TIL is hereby demonstrated.

## Results

### Functional CRISPR/Cas9 screens identify SOCS1 as a top target constraining in vitro expansion of TIL and in vivo infiltration of transferred CD8^+^ T cells in syngeneic solid-tumor models.

**Figure 1:**
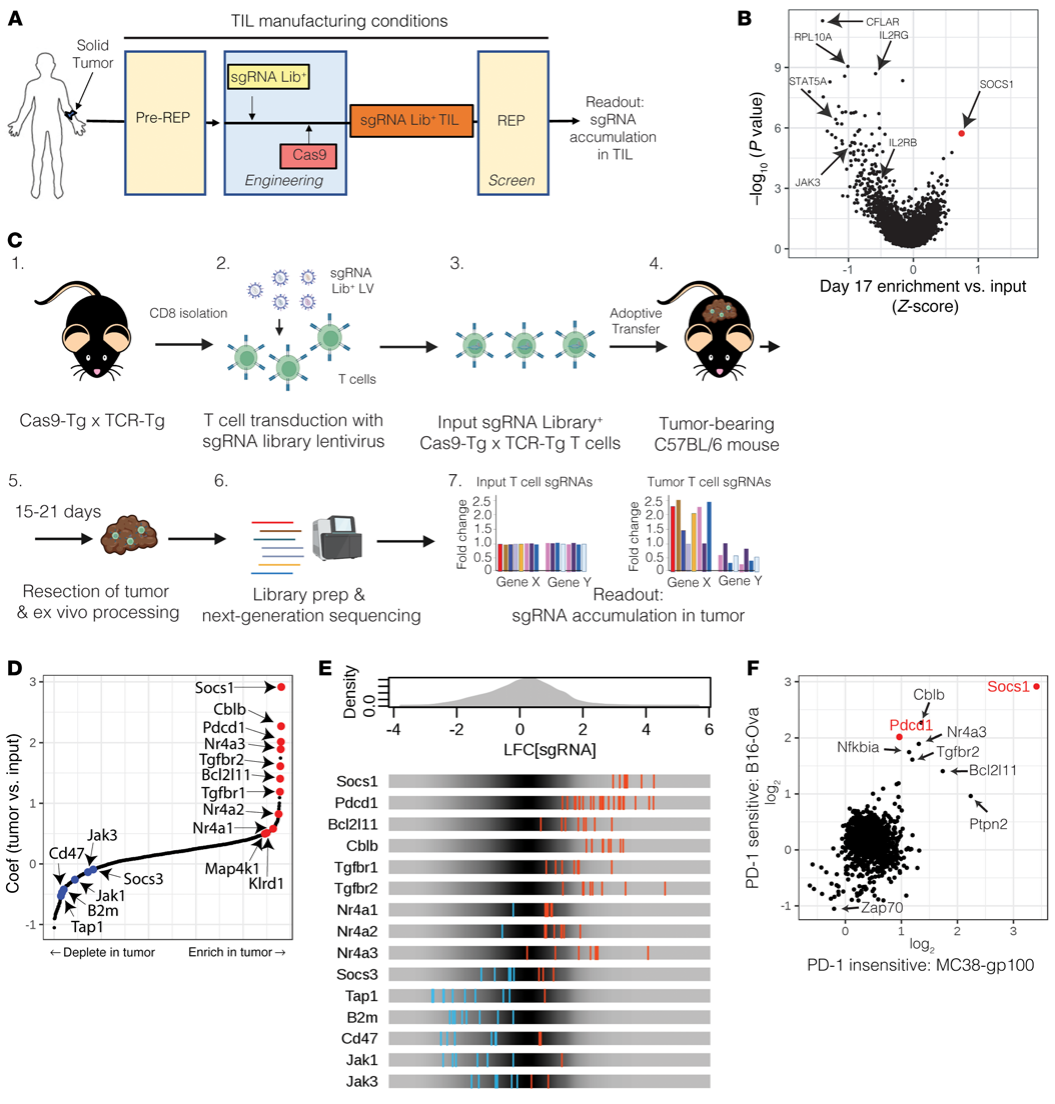
Functional CRISPR/Cas9 screens identify SOCS1 as a top target constraining in vitro expansion of TILs and in vivo infiltration of transferred CD8^+^ T cells in syngeneic solid-tumor models. (**A**) Experimental schematic depicting a CRISPR screen performed on the in vitro expansion of human melanoma TILs under conditions used to manufacture for therapeutic use. Following pre-REP, a sgRNA library was introduced by lentiviral transduction with TILs, then engineered by introduction of Cas9 by electroporation. A REP was then initiated in the presence of allogeneic iPBMC feeders, OKT3, and IL-2. The distribution of sgRNAs was compared prior to and following the REP to identify targets enhancing TIL accumulation. (**B**) SOCS1 is the top target enhancing the accumulation of TIL under REP conditions. (**C**) Experimental schematic depicting the workflow of in vivo CRISPR screens using Cas9-Tg × TCR-Tg (OT1 or pmel) CD8^+^ T cells. CD8^+^ T cells were activated and transduced to express a sgRNA library, with Cas9-Tg × TCR-Tg T cells transferred into mice bearing 100 mm^3^ tumors on the flank. Either 14 or 21 days following transfer, tumors were harvested and the sgRNA distribution of T cells within tumors analyzed and compared with the input population of T cells. (**D**) MAGeCK-MLE identifies SOCS1 as a top target enhancing OT1 T cell infiltration into B16-OVA tumors 14 days following transfer. (**E**) Enrichment or depletion patterns of sgRNAs targeting known genes by tumor OT1s in comparison with input OT1s. (**F**) MAGeCK-MLE identifies SOCS1 as a top target enhancing the infiltration of pmel CD8^+^ T cells 14 days after transfer into the MC38-gp100 solid-tumor model found to be refractory to inhibition of PD-1.

To identify targets that can enhance TIL function, we performed large-scale in vitro and in vivo CRISPR/Cas9 screens. The manufacture of TIL for therapeutic use occurs in a multistep process involving surgical resection of tumor, extraction of TIL from tumor fragments in the presence of IL-2 in a step known as “pre-REP” (with REP indicating rapid expansion phase), followed by expansion of TIL in the presence of IL-2, irradiated PBMC feeders, and agonistic anti-CD3 OKT3 antibody in the REP (29–32). As attaining the appropriate dose level of TIL can be a challenge, especially with heavily pretreated patients, CRISPR was performed on TILs derived from a single metastatic melanoma donor to identify gene targets enhancing expansion under REP conditions. A barcoded sgRNA library encompassing a curated list of 5,137 genes involved in T cell function, all predicted cell-surface receptors (CSRs), and genes found to be expressed within immune cells and peripheral blood was introduced by lentiviral transduction into pre-REP TILs together with Cas9. Engineered sgRNA Lib^+^ TILs were then expanded under REP conditions (Figure 1A). In this screen, SOCS1 was identified as the top hit driving the expansion of TIL in REP conditions (FDR-adjusted *P* = 4.03 × 10^–4^) (Figure 1B and Supplemental Table 1; supplemental material available online with this article; https://doi.org/10.1172/JCI163096DS1). Confirming screen robustness, we observed recovery of 85% library sgRNAs as well as depletion of sgRNAs targeting essential genes, including strong depletion of RPL10A, as well as multiple members of the IL-2 receptor signaling complex, including IL-2RG, STAT5A, JAK3, and IL-2RB (Supplemental Data 1 and Figure 1B), consistent with the dependency of TILs on IL-2 survival signals during REP.

To identify targets that enhance the ability of CD8^+^ T cells to infiltrate, accumulate, and persist in solid tumors in vivo, we crossed Cas9 transgenic mice (33) with OT1 TCR-Tg mice with the Cas9-Tg × OT1 CD8^+^ T cells recognizing OVA peptide (34). We conducted a CRISPR screen in the B16-OVA model, found to be sensitive to transferred OT1 T cells in which the *Pdcd1* gene encoding PD-1 has been inactivated using CRISPR/Cas9 (Supplemental Figure 1A). Two barcoded sgRNA libraries were introduced into Cas9-Tg × OT1 T cells covering a curated list of 369 T cell genes as well as 1,004 predicted CSRs. Following transfer of sgRNA^+^ Cas9-Tg × OT1 T cells into B16-OVA tumor-bearing mice, the distribution of sgRNAs present within the transferred cells infiltrating tumor was evaluated 14 or 21 days later (Figure 1C). We observed recovery of 97% of sgRNAs from tumor samples (Supplemental Data 2) and strong enrichment of sgRNAs targeting the *Pdcd1* gene (FDR-adjusted *P* < 1 × 10^–16^). Confirming screen robustness, sgRNAs targeting genes required for T cell function were depleted from the tumor, including *Tap1*, *B2m*, *CD47*, *Jak1*, and *Jak3* (Figure 1, D and E). In addition, our screen showed enrichment of sgRNAs targeting other genes encoding negative regulators of T cell function, including *Bcl2l11*, *Cblb*, both *Tfgbr1* and *Tgfbr2*, *Kldr1*, and all 3 members of the *Nr4a* family (Figure 1, D and E). Notably, sgRNAs targeting the *Socs1* gene encoding SOCS1 emerged as the strongest targets driving enhanced CD8*^+^* T cell infiltration within tumors (FDR-adjusted *P* < 1 × 10^–16^), greater than sgRNAs targeting *Pdcd1* (Figures 1, D and E, and Supplemental Table 2).

The highest unmet medical need for the treatment of solid tumors is in the PD-1 refractory setting. We therefore performed an additional in vivo CRISPR/Cas9 screen using pmel CD8*^+^* T cells in an MC38-gp100 tumor model found to be insensitive to treatment by *Pdcd1*-inactivated pmel T cells (35) The screen again performed robustly, with 82% of the sgRNAs recovered (Supplemental Data 3) and with sgRNAs targeting the *Pdcd1* gene displaying no enrichment within tumors (Figure 1F). In contrast, *Socs1* again scored as the top T cell target in this screen (FDR-adjusted *P* < 1 × 10^–16^) (Figure 1F). In this particular screen, other T cell targets that enriched in the OT1/B16-OVA screen did not demonstrate as strong enrichment, including *Cblb*, *Bcl2l11*, *Tgfbr2*, *Nr4a3*, and *Nfkbia* (Figure 1F and Supplemental Table 2).

Collectively, these data identify SOCS1 as a target that, upon CRISPR/Cas9-mediated inactivation, enhances the ability of TILs to expand under manufacturing conditions and to drive the infiltration and accumulation of tumor-specific CD8^+^ T cells within solid tumors in an in vivo setting.

### Inactivation of SOCS1 by CRISPR/Cas9 in transferred CD8^+^ T cells drives enhanced efficacy in syngeneic mouse models with durable persistence as Tcm cells.

**Figure 2:**
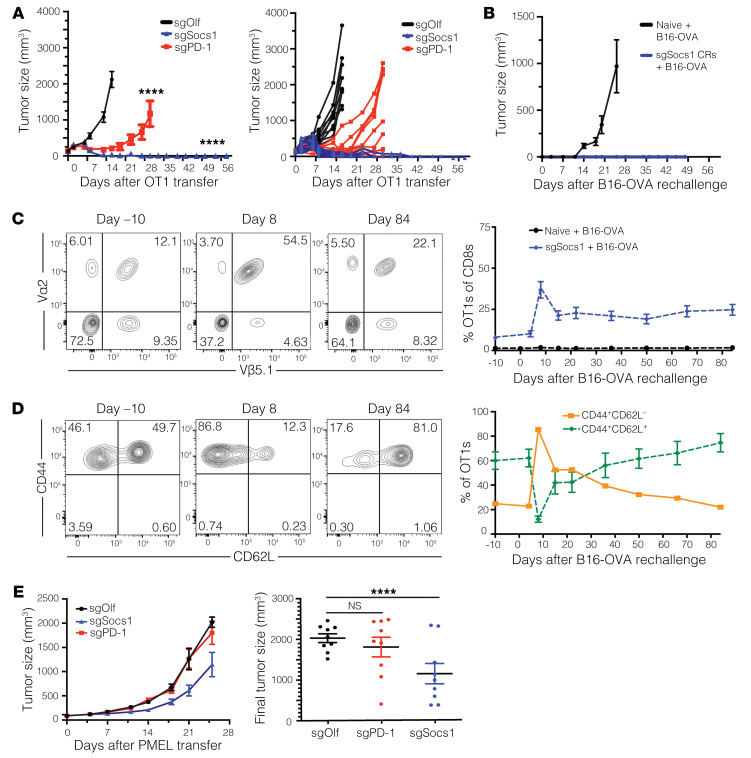
Inactivation of SOCS1 by CRISPR/Cas9 in transferred CD8^+^ T cells drives enhanced efficacy in syngeneic mouse models with durable persistence as Tcm cells. C57BL/6 mice bearing 100 mm^3^ B16-OVA tumors on the flank were treated with 3 × 10^6^ OT1 CD8^+^ T cells engineered to inactivate either OLF1 (sgOlf), PD-1 (sgPD-1), or SOCS1 (sgSocs1). Results of statistical analysis depicted between sgOlf versus sgPD-1 and sgOlf versus sgSocs1. (**A**) Tumor growth curves of each group over time are depicted. (**B**) Mice treated with sgSocs1 OT1s undergoing complete tumor rejection were rechallenged with B16-OVA tumor cells 61 days following initial transfer, with 10 naive mice included as controls. Tumor growth of the indicated treatment groups is depicted. (**C**) The frequency of sgSocs1 OT1s as peripheral blood CD8^+^ T cells prior to (day –10) and following (days 8 through 84) B16-OVA rechallenge. FACS plots are depicted, with OT1s defined as CD8^+^Va2^+^Vβ5.1^+^cells. (**D**) CD44^+^CD62L^+^ and CD44^+^CD62L^–^ phenotypes of sgSocs1 OT1s in **C** prior to (day –10) and following B16-OVA rechallenge (days 8 and 84) were quantified by FACS and depicted. (**E**) C57BL/6 mice bearing MC38-gp100 tumors with a median size of 100 mm^3^ were treated with 7 × 10^6^ pmel CD8^+^ T cells inactivated with either SOCS1 (sgSocs1), PD1 (sgPD-1), or OLF1 (sgOlf). Tumor growth curves depicted by treatment group on graph on the left, and final tumor size in individual mice depicted on graph on the right. Results of 2-way ANOVA were used to determine statistical significance between treatment groups in **A** and **E**. ****P < 0.0001. Data in **A**–**D** are representative of 2 independent experiments.

We next evaluated the impact of inactivating SOCS1 on the antitumor function and memory formation of transferred CD8^+^ T cells. OT1 T cells were edited using CRISPR/Cas9 ribonucleoproteins (RNPs) targeting either *Socs1* (sgSocs1), *Pdcd1* (sgPD-1), or *Olf1a* (sgOlf) as a negative control and were transferred into mice bearing established 100 mm^3^ B16-OVA tumors. Editing efficiencies for target genes were 82% for sgPD-1, 92% for sgSocs1, and 80% for sgOlf. Mice receiving sgSocs1-edited OT1 T cells displayed clearance of tumors, with 10 of 10 mice undergoing complete rejection (CR) of tumor in comparison with the inadequate tumor control demonstrated by mice receiving sgPD-1 or sgOlf OT1s (Figure 2A). sgSocs1 OT1–treated mice undergoing a CR were rechallenged with B16-OVA tumors 76 days following initial transfer and completely rejected secondary tumor challenge (Figure 2B). The persistence and phenotype of sgSocs1 OT1s were tracked longitudinally in the peripheral blood of CR mice both immediately prior to and following B16-OVA rechallenge. Following initiation tumor rejection, sgSocs1 OT1s made up 7% of blood CD8^+^ T cells (Figure 2C), with a CD44^+^CD62L^+^ phenotype reflecting Tcm cells (Figure 2D). Following rechallenge, sgSocs1 OT1s expanded to occupy 37% of total blood CD8^+^ cells and displayed a CD44^+^CD62L^–^ T effector memory (Tem) cell phenotype (Figure 2D), followed by contraction to a new baseline of 24% of total CD8^+^ T cells which persisted out to 160 days until the termination of the experiment, and displaying a CD44^+^CD62L^+^ Tcm phenotype (Figure 2D).

To evaluate SOCS1 inactivation in transferred CD8^+^ T cells in a syngeneic model refractory to PD-1 inactivation, sgOlf, sgSocs1, or sgPD-1 pmel cells were adoptively transferred into mice bearing established 100 mm^3^ MC38-gp100 tumors. Mice receiving sgSocs1 pmels showed controlled tumor growth, with hosts receiving sgPD-1 edited or sgOlf pmels showing no impact on tumor growth (Figure 2E). To quantify how inactivation of Socs1 in T cells improves antitumor potency in an in vivo setting, we performed a relative potency study in which the antitumor activity of a dose titration of sgOlf and sgSocs1 OT1s was evaluated. A dose of 4.1 × 10^6^ sgSocs1 OT1s matched the antitumor-response profile generated by 41 × 10^6^ sgOlf-edited OT1s, suggesting that inactivation of *Socs1* leads to an estimated 10-fold antitumor potency increase in OT1 T cells (Supplemental Figure 2A). In the 41 × 10^6^ dose-level cohort where both sgOlf and sgSocs1 treatment groups showed similarly strong antitumor activity, sgSocs1 OT1s were present at a 5-fold greater frequency within the peripheral blood in comparison with sgOlf OT1–treated mice, establishing that inactivation of SOCS1 enhances the overall accumulation of transferred T cells even in similarly responding mice (Supplemental Figure 2B). sgSocs1 OT1s additionally demonstrated enhanced accumulation depicted as CD44^+^CD62L^+^ Tcm cells versus sgOlf OT1s (Supplemental Figure 2C).

To conclude, inactivation of SOCS1 in CD8^+^ T cells leads to a robust increase in antitumor activity in vivo, including activity in models in which inactivation of PD-1 on CD8^+^ T cells has no effect. SOCS1 inactivation led to an increase in the frequency of transferred T cells detectable within the peripheral blood, with these cells possessing a Tcm phenotype and remaining prominent within the host as far out as 160 days following transfer.

### Inactivation of SOCS1 in transferred CD8^+^ T cells enhances accumulation of CD44^+^CD62L^+^ Tcm cells within lymphoid organs and Slamf6^–^CD39^+^PD-1^hi^ Tex cells in tumors while depleting intratumoral Tregs.

**Figure 3:**
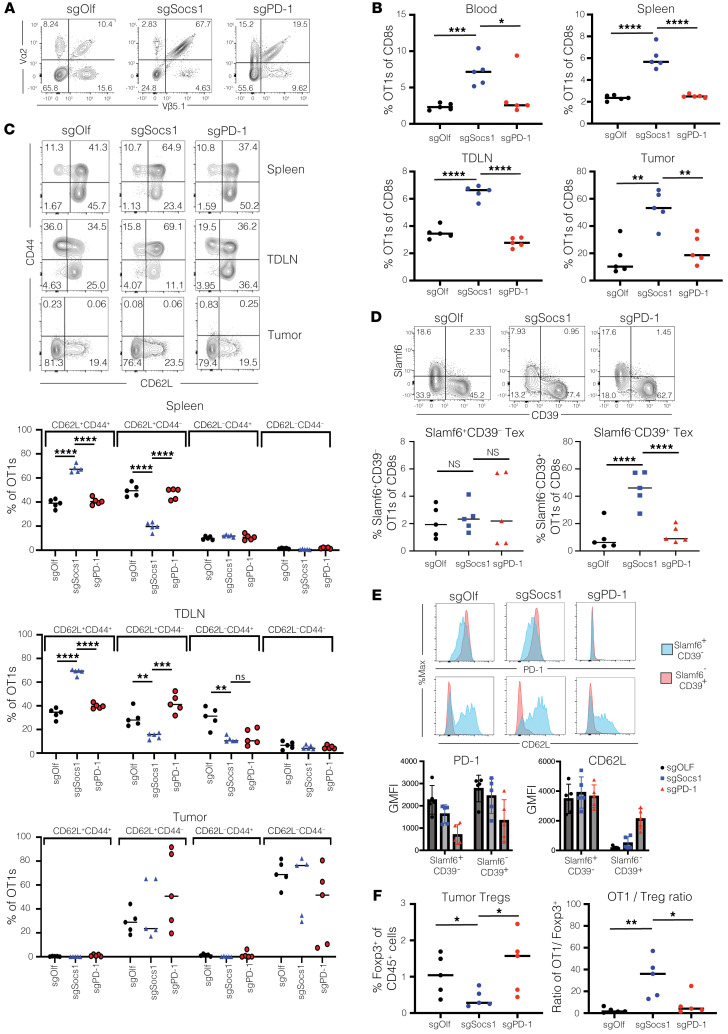
Inactivation of SOCS1 in transferred CD8^+^ T cells enhances their accumulation as CD44^+^CD62L^+^ Tcm cells within lymphoid organs and Slamf6^–^CD39^+^PD-1^hi^ Tex cells in tumors while depleting intratumoral Tregs. C57BL/6 mice bearing 100 mm^3^ B16-OVA tumor cells bearing a median size of 100 mm^3^ were treated with 3 × 10^6^ SOCS1 (sgSocs1), PD1 (sgPD-1), or OLF1 (sgOlf) engineered OT1s. (**A**) OT1 frequency in tumors 7 days following transfer was determined by quantifying the frequency of CD8^+^Va2^+^Vβ5.1^+^ cells within the CD8^+^ T cell compartment, with data from representative mouse shown. (**B**) Frequency of CD8^+^Va2^+^Vβ5.1^+^ cells in the blood, spleen, TDLNs, and tumor between treatment groups. (**C**) Frequency of OT1s between treatment groups expressing CD62L and/or CD44 are depicted from TDLNs, spleen, and tumor. (**D**) Expression patterns of Slamf6 and CD39 by OT1s from tumor depicted. (**E**) Expression of PD-1 or CD62L protein expressed by either Slamf6^+^CD39^–^ or Slamf6^–^CD39^+^ intratumoral OT1s from each treatment group is depicted from a representative mouse in the top panel, with compiled data from individual mice shown in the bottom panel. (**F**) Frequency of CD4^+^Foxp3^+^ cells within the TME was quantified in relation to total CD45^+^ cells (left panel) and as a ratio of OT1s to Tregs (right). Each symbol reflects an individual mouse. *P < 0.05; **P < 0.01; ***P < 0.001; ****P < 0.0001, unpaired, 2-tailed Student’s *t* test between the indicated comparator groups. For **B**–**D** and **F**, *P* values were adjusted for multiple testing correction using the Benjamini-Hochberg method.

We next sought to better understand how SOCS1 inactivation affected CD8^+^ T cell function within tumors and lymphoid organs during the acute phase of the antitumor response. We evaluated the frequency and phenotype of transferred sgSocs1, sgPD-1, and sgOlf OT1 T cells 7 days following transfer, with editing efficiencies for target genes 82% for sgPD-1, 95% for sgSocs1, and 80% for sgOlf. We confirmed our initial screen observation that sgSocs1 OT1s enriched to a greater degree within tumor, with sgSocs1 OT1s making up 53% of total CD8^+^ T cells versus 16% of sgOlf and 23% of sgPD-1 OT1s (Figure 3, A and B). A similar enrichment of sgSocs1 OT1s was observed in the peripheral blood, spleen, and tumor-draining inguinal lymph nodes (TDLN) (Figure 3B). In evaluating the phenotype of transferred OT1s, a near doubling in frequency of CD44^+^CD62L^+^ Tcm OT1 cells was observed in the spleen and TDLNs in the sgSocs1 treatment group in comparison with sgOlf and sgPD-1 OT1s (Figure 3C). Conversely, upon evaluating OT1s infiltrating tumor, a heterogenous pattern of CD62L expression was observed, with no detectable expression of CD44 (Figure 3C). Tumor-infiltrating OT1s were further assessed to discern the Tex differentiation state. In the T cell exhaustion differentiation continuum, TCF-1–expressing stem-like Tex^prog^ cell subsets can be identified using Slamf6 (36). Tex^prog^ cells undergo clonal expansion and differentiation into Tex subsets specializing in effector function and terminal differentiation, and Slamf6 expression is lost and CD39 expression induced, with PD-1 increasing in expression (37, 38). Tumor-infiltrating OT1s across treatment groups could be divided into Slamf6^+^CD39^–^ Tex^prog^ and Slamf6^–^CD39^+^ cells encompassing Tex^int^, Tex^eff^, and terminally differentiated Tex^term^ CD8^+^ subsets (Figure 3D). No impact was found on the frequencies of Slamf6^+^CD39^–^ Tex^prog^ subsets between groups (Figure 3D). However, inactivation of SOCS1 in sgSocs1 OT1s drove a marked enhancement in the accumulation of the Slamf6^–^CD39^+^ population in comparison with sgOlf and sgPD-1 treatment groups (Figure 3D). Additional phenotypic analysis confirmed expression of CD62L by the Slamf6^+^CD39^–^ population in comparison with the Slamf6^–^CD39^+^ population and with Slamf6^–^CD39^+^ OT1s conversely expressing higher levels of PD-1 protein, apart from the sgPD-1 OT1 treatment group, which was confirmed to not express detectable levels of PD-1 protein (Figure 3E). To summarize, inactivation of SOCS1 in transferred CD8^+^ T cells led to (a) enhanced accumulation in blood, spleen, TDLNs, and tumor; (b) increased accumulation as CD44^+^CD62L^+^ Tcm cells in the spleen and TDLNs, and (c) no impact on the frequency of Slamf^+^CD39^–^PD-1^med^ Tex^prog^ cells while (d) markedly increasing the accumulation of Slamf^–^CD39^+^PD-1^hi^ cells encompassing Tex^int^, Tex^eff^, and CD8 subsets.

In assessing the impact of SOCS1 and PD-1 inactivation in transferred CD8^+^ T cells on the cellular composition of tumor, we observed depletion of Foxp3^+^ Tregs relative to sgOlf OT1s in mice treated with sgSocs1 OT1s, but not sgPD-1 OT1–treated animals. This resulted in intratumoral OT1/Treg ratios of 32-fold, with sgSocs1 OT1s in comparison with 2.6-fold with sgOlf OT1s and 8-fold with sgPD-1 OT1s (Figure 3F). Of note, the mean tumor volumes of sgOlf-, sgPD-1–, and sgSocs1-treated groups on the day of tumor harvest were 394 mm^3^ SEM ± 35, 210 mm^3^ SEM ± 110, and 287 mm^3^ SEM ± 81, respectively, suggesting that the depletion of Tregs in the sgSocs1 treatment group is not driven by stronger efficacy and smaller tumor size. We also assessed the impact of treatment groups on intratumoral innate cell-population frequencies (39). Populations evaluated included CD45^+^CD11b^+^Ly6C^+^Ly6G^+^ neutrophils; CD45^+^CD11b^+^Ly6C^+^Ly6G^–^MHCII^+^F4/80^–^ monocytic myeloid–derived suppressor cells (mMDSCs); CD45^+^CD11b^+^Ly6C^med^Ly6G^–^MHCII^+^F4/80^+^ tumor-associated macrophages (TAMs), including CD11c^–^ TAM1 and CD11c^+^ TAM2 subsets; and CD45^+^CD11b^+^Ly6C^–^MHCII^+^ CD11c^+^ classical DCs (cDCs), including CD103^+/–^ cDCs and CD11b^+^NKp46^+^ NK cells (Supplemental Figure 3, A and B). The sgSocs1 treatment was found to increase the presence of neutrophils and decrease the presence of CD103^+^ cDCs in tumor (Supplemental Figure 3B). These results together with the observed depletion of intratumoral Tregs driven by sgSocs1 OT1s indicate that inactivation of SOCS1 in transferred CD8^+^ T cells remodels tumor by in part reducing the frequency of immunosuppressive cell subsets known to impede T cell antitumor function.

### SOCS1 is a key checkpoint in the accumulation of Tex^int^ and Tex^eff^ cells from Tex^prog^ subsets within tumors with mechanisms distinct from that of PD-1.

**Figure 4:**
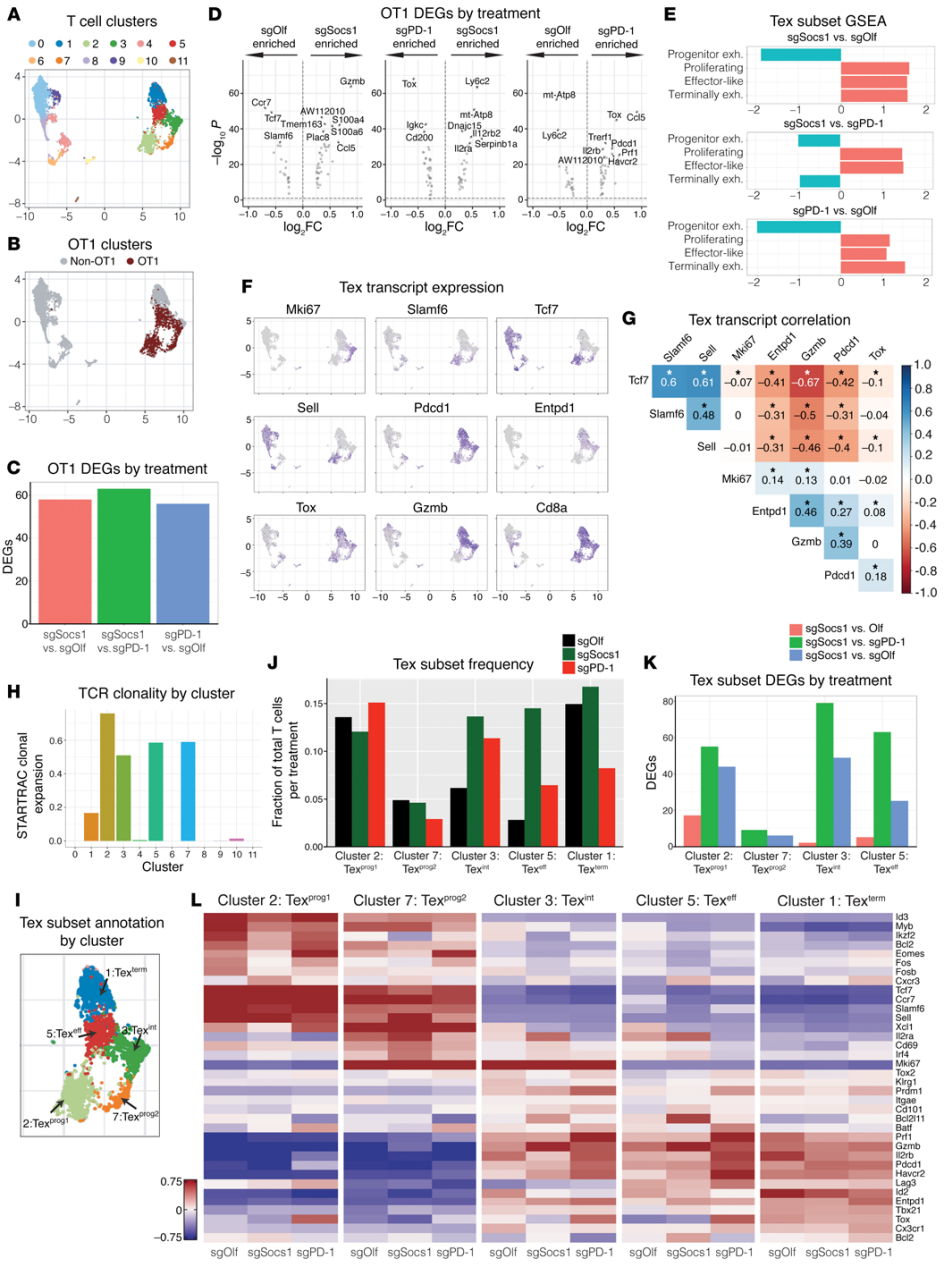
SOCS1 is a key checkpoint in the accumulation of Tex^int^ and Tex^eff^ cells from Tex^prog^ subsets within tumors with mechanisms distinct from PD-1. C57BL/6 mice bearing 100 mm^3^ B16-OVA tumor cells with a median size of 100 mm^3^ were treated with 3 × 10^6^ SOCS1 (sgSocs1), PD1 (sgPD-1), or OLF1 (sgOlf) engineered OT1s, with editing efficiencies for target genes 82% for sgPD-1, 95% for sgSocs1, and 80% for sgOlf. scRNA-Seq was performed on CD45^+^ cells isolated from the TME from each treatment group. (**A**) UMAP visualization of T cell clusters. (**B**) Projection of OT1s onto T cell clusters based on TCR sequencing. (**C**) Bar plot of treatment DEGs between treatment-group OT1s, adjusted *P* < 0.1, abs(avg_log_2_FC ≥ 0.25). (**D**) Pseudobulk analysis on OT1s, with DEGs between treatment groups depicted. (**E**) GSEA by projecting pseudobulk DEGs from between indicated treatment groups in **D** onto Miller et al. Tex subset gene signatures (41). (**F**) UMAP visualization depicting the expression of indicated transcripts by T cell clusters (**G**) Correlogram between indicated transcripts (*P < 0.001). (**H**) STARTRAC TCR clonal expansion by T cell cluster. (**I**) Tex subset annotation by cluster. CD8, terminally differentiated Tex subsets. (**J**) Tex subset frequency by treatment group. Clusters 2, 7, 3 and 5 reflect OT1 frequencies, with cluster 1 containing non-OT1 cells and included for reference. (**K**) Number of DEGs within each Tex subset as indicated and between depicted treatment groups. (**L**) Heatmap of Tex subset–defining transcripts by subset and by treatment group.

To understand in greater detail how inactivation of SOCS1 affects the state of transferred CD8^+^ T cells within the tumor, single-cell RNA–Seq (scRNA-Seq) (10x Genomics) was performed on CD45^+^ bead-selected cells isolated from B16-OVA tumors harvested 7 days following the transfer of either sgSocs1, sgPD-1, or sgOlf OT1s. Across all treatment groups, 18,794 independent transcriptomes were captured, with expression signatures revealing the presence of major immune cell types, including macrophages, DCs, NK cells, B cells, and T cells (Supplemental Figure 4A). Differences in overt cell-type populations between treatment groups were minimal, with the exception being an enrichment of B cells in the sgPD-1–treated group (Supplemental Figure 4B). Analysis of the CD3^+^ T cell subpopulation comprising 8,123 independent transcriptomes revealed 11 distinct clusters (Figure 4A, with Supplemental Figure 4C showing depicted cluster cell counts), as defined by T cell cluster marker genes (Supplemental Figure 4D). The use of 5′ scRNA-Seq permitted detection of OT1 TCR α and β chain sequences within the annotated CD3^+^ clusters, with 2,377 OT1s identified (sgOlf, 674; sgSocs1,1187; and sgPD-1, 516 OT1s detected per treatment group, respectively) and found to localize within 4 T cell clusters: 2, 3, 5, and 7 (Figure 4B). Detection of discrete OT1s allowed for pairwise comparison of gene expression signatures between treatment groups, with pseudobulk RNA-Seq analysis used to identify differentially expressed genes (DEGs), both induced (DEG^up^)and depleted (DEG^down^) (Figure 4C). Similar numbers of absolute DEGs were observed in all treatment group comparisons (Supplemental Figure 4E), with the high number of DEGs in the sgSocs1 versus sgPD-1 comparison indicating distinct mechanistic differences in how inactivation of SOCS1 or PD-1 affect CD8^+^ T cell transcriptional states. In the sgSocs1 versus sgOlf comparison, *Gzmb* transcripts were the highest DEG^up^, with *Ccr7*, *Tcf7*, and *Slamf6* identified as the strongest DEG^down^ (Figure 4D). When comparing the sgPD-1 OT1s to either sgSocs1 or sgOlf OT1s, *Tox* transcripts encoding for TOX, a transcription factor and master regulator of T cell exhaustion (40), were among the strongest DEG^up^ (Figure 4D). Gene set enrichment analysis (GSEA) was performed by projecting DEGs from pairwise treatment group comparisons onto published gene expression signatures described by Miller et al. to define progenitor, proliferating, effector-like, and terminally exhausted Tex subsets transcriptionally and functionally (41). We found that both sgSocs1 and sgPD-1 OT1s were enriched in proliferating, effector-like, and terminally exhausted Tex expression signatures, while they were depleted in progenitor signatures when compared with sgOlf OT1s (Figure 4E). Conversely, when comparing sgSocs1 versus sgPD-1 OT1s, sgSocs1 was enriched for proliferating and effector-like and depleted of both progenitor and terminally exhausted Tex signatures (Figure 4E). These results together with the observed induction of *Tox* transcripts by sgPD-1 OT1s highlight a mechanistic distinction between the impact of sgSocs1 and that of sgPD-1 on the transcriptional state of transferred CD8^+^ T cells.

Our FACS analysis of tumor-infiltrating OT1s found that inactivation of SOCS1 in transferred CD8^+^ T cells did not affect Slamf6^+^CD39^–^PD-1^med^ Tex^prog^ cell frequency while enhancing the accumulation of Slamf6^–^CD39^+^PD-1^hi^ cells containing Tex^int^, Tex^eff^, and CD8^+^ T cells (Figure 3D). We next sought to query CD8^+^ Tex states encompassed within these T cell clusters in more detail. To ensure that our analysis captured all T cell clusters responding to cognate tumor antigen and including non-OT1 endogenous T cells, we identified T cell clusters with high TCR clonality and thus undergoing clonal expansion using STARTRAC (42). We observed expected strong TCR clonality within the 4 OT1-enriched clusters and identified cluster 1 as also characterized by high TCR clonality, even with few OT1s present (Figure 4H). We focused our analysis of Tex subsets on these 5 clusters hereafter. These clusters demonstrated ubiquitous expression of *CD8a* transcripts with heterogenous expression of Tex-state defining transcripts. Cluster 1 was represented by *Pdcd1*, *Entpd1*, *Tox*, and *Gzmb* transcripts; cluster 2 was represented by *Slamf6*, *Tcf7*, and *Sell*; cluster 3 was represented by *Mki67*, *Pdcd1*, and *Gzmb*; cluster 5 was represented by *Pdcd1*, *Entpd1*, and *Gzmb*; and cluster 7 was represented by *Mki67*, *Slamf6*, *Tcf7*, and *Sell* (Figure 4F). Strong positive correlation of transcript expression was observed between *Tcf7*, *Slamf6*, and *Sell* and between *Entpd1*, *Gzmb*, and *Pdcd1*, with *Tcf7*, *Slamf6*, and *Sell* in turn all showing strong inverse correlation with *Entpd1*, *Gzmb*, and *Pdcd1* transcripts (Figure 4G). The inverse correlation between *Slamf6* and *Entpd1* transcripts, which encode for Slamf6 and CD39 proteins, respectively, is consistent with our observations shown in Figure 3D, in which Slamf6^+^CD39^–^ and Slamf6^–^CD39^+^ populations could be clearly demarcated.

To align Tex cell states to each cluster undergoing clonal expansion, we compared cluster expression signatures to published RNA-Seq and scRNA-Seq data sets derived from chronic viral infection and tumor settings and used to define exhausted T cells subsets based on transcriptional and functional profiles (41, 43). Each cluster was projected onto gene signatures previously used to define progenitor Tex cells, including subset 1 (Tex^prog1^) and subset 2 (Tex^prog2^), Tex^int^ cells, Tex^eff^ cells, and Tex^term^ CD8^+^ T cells (Supplemental Figure 4F). We were able to discern the continuum of the Tex differentiation pathway within our 5 identified clusters (Figure 4, I and L) (41, 43, 44) and identified the Tex^prog1^ subset as quiescent *Slamf6^+^Tcf7^+^* cells (cluster 2) and the Tex^prog2^ subset as proliferating *Mki67^+^Slamf6^+^Tcf7^+^* cells (cluster 7), with both clusters reflecting TCF-1–expressing stem-like progenitor Tex cells. A population of proliferating Tex^int^ *Mki67^+^Gzmb^+^Entpd1^+^* cells (cluster 3) and quiescent Tex^eff^ *Mki67^–^Gzmb^+^Entpd1^+^* cells (cluster 5) reflecting Tex cells with effector capabilities characterized by expression of *Gzmb* transcripts were also identified, as well as a population of quiescent *Gzmb^+^Entpd1^+^Tox^+^* cells (cluster 1) reflecting Tex^term^ cells as characterized by high expression of *Tox* transcripts. Of note, few OT1s were present in the Tex^term^ cluster. Tex^prog1^, Tex^eff^, and Tex^term^ clusters were found to be enriched for TCR activation pathway gene signatures and with Tex^prog2^ and Tex^int^ conversely showing depletion, correlating with the quiescent state of the former and the proliferating state of the latter Tex subsets (Supplemental Figure 4G) (45). The impact of SOCS1 or PD-1 inactivation on Tex subsets was next evaluated. SOCS1 or PD-1 inactivation had no impact on overall frequencies of either Tex^prog1^ or Tex^prog2^ subsets reflecting TCF1-expressing Tex^prog^ subsets (Figure 4J). Given heightened expression of *Slamf6* and lack of expression of *Entpd1* transcripts by Tex^prog1^ and Tex^prog2^ subsets, this is consistent with our observation that treatment did not affect frequencies of Slamf6^+^CD39^–^ OT1s (Figure 3D). However, inactivation of SOCS1 in the sgSocs1 OT1 group drove a statistically significant enrichment of both Tex^int^ (*P* = 7.67 × 10^–17^) and Tex^eff^ subsets (*P* = 1.7 × 10^–62^) reflecting proliferating and quiescent Tex subsets with effector capabilities (Figure 4J). As Tex^int^ and Tex^eff^ subsets do not express *Slamf6*, yet express *Entpd1* transcripts, this finding is also consistent with our observed increase in frequency of Slamf6^–^CD39^+^ OT1s in the sgSocs1 treatment group. CD4^+^Foxp3^+^ Tregs localized to cluster 10 (Supplemental Figure 4H), with this cluster depleted in the sgSocs1 treatment group (*P* = 1.33 × 10^–05^), consistent with our FACS data (Figure 3F). To determine how treatment groups affected gene expression signatures within discrete Tex clusters, we examined DEGs between treatments within Tex subsets (Figure 4K) and evaluated the expression patterns of a curated list of transcripts involved in defining the Tex state (Figure 4L). Interestingly, pairwise comparison of the sgOlf versus sgSocs1 yielded few DEGs within each Tex cluster (Figure 4K and Supplemental Figure 4J). For Tex subset DEGs in the sgPD-1 versus sgOlf and sgSocs1 versus sgPD-1 comparisons, a far greater overall number of DEGs were identified (Figure 4K), with *Tox* again a top DEG^up^ in the sgPD-1 group across multiple Tex subsets (Figure 4L and Supplemental Figure 4J). We note that the detection of transcripts aligning to the *Pdcd1* gene in the sgPD-1 group (Figure 4L), despite an editing efficiency of 82% in sgPD-1 OT1s with a sgRNA targeting exon 2 of the *Pdcd1* gene, is likely due to our use of 5′ scRNA-Seq permitting detection of transcribed yet edited transcripts from the *Pdcd1* gene.

These scRNA-Seq data confirm and extend our FACS analysis in demonstrating how inactivating SOCS1 affects the trajectory of transferred CD8^+^ T cells as Tex subsets within tumors in comparison with PD-1. Tex^int^ and Tex^eff^ cells are known to differentiate from Tex^prog^ subsets (46), with our data suggesting that SOCS1 serves as a key checkpoint in the accumulation of Tex^int^ and Tex^eff^ cells from Tex^prog^ cells. Inactivation of SOCS1 had little impact on the transcriptional states of Tex subsets, with the most pronounced impact of SOCS1 inactivation being the overt increase of Tex^int^ and Tex^eff^ subsets exhibiting high expression of *Gzmb* transcripts. The impact of SOCS1 inactivation on transferred CD8^+^ T cells is mechanistically distinct from that of PD-1, since while inactivation of PD-1 enriched for Tex^eff^ subsets, albeit to a lesser degree than inactivation of SOCS1, it also enriched for a terminally exhausted gene signature, including heightened expression of *Tox* transcripts.

### A CRISPR tiling screen in primary human T cells identifies highly potent sgRNAs for therapeutic use targeting the SH2 domain of SOCS1.

**Figure 5:**
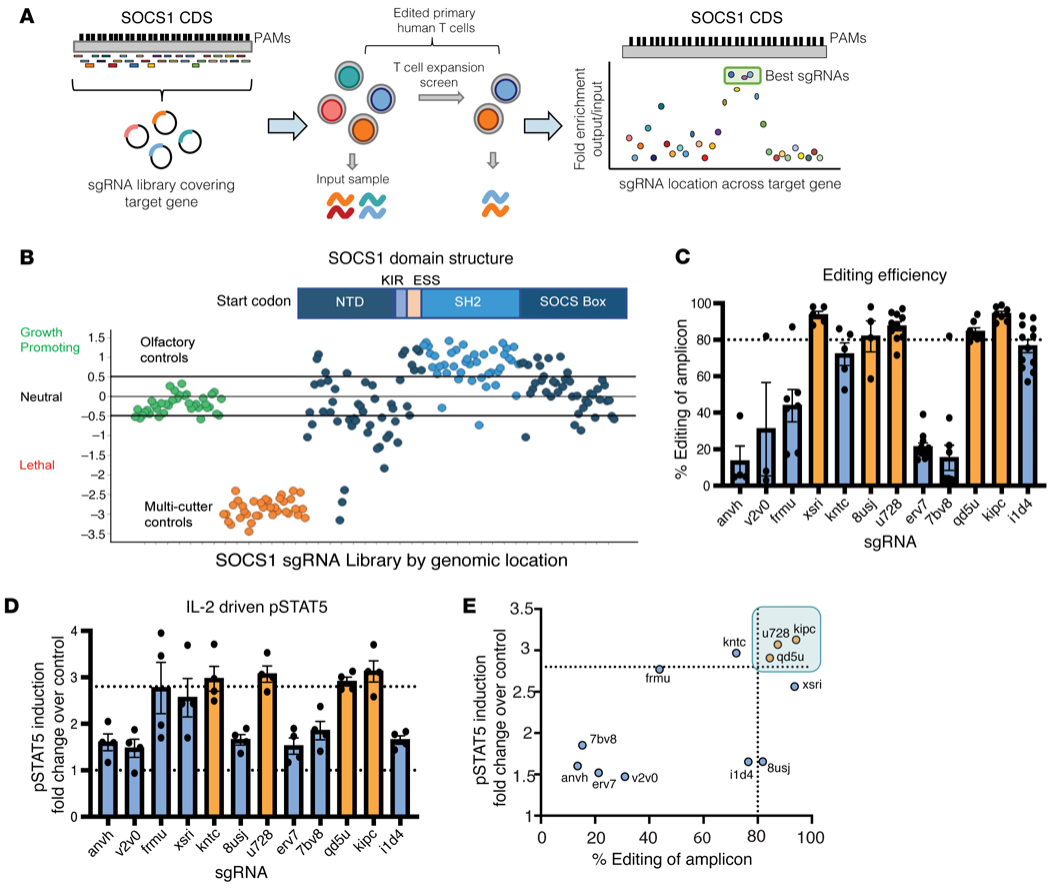
A CRISPR tiling screen in primary human T cells identifies highly potent sgRNAs for therapeutic use targeting the SH2 domain of SOCS1. (**A**) Experimental schematic of the CRISPR tiling screen for discovering potent SOCS1 sgRNAs. Following in silico removal of sgRNAs predicted to target multiple sites in the genome, a sgRNA library targeting every possible Cas9 cut site of the SOCS1 CDS based on the trinucleotide NGG PAM sequence together with controls was introduced by lentiviral transduction into activated primary human T cells with IL-2. Following introduction of Cas9, sgRNA Lib^+^ T cells were expanded in the presence of IL-2, with the distribution of sgRNAs following expansion evaluated and compared with input. (**B**) SOCS1 CRISPR tiling screen results. sgRNAs targeting the olfactory genes are in green, genome multicutters in orange, and sgRNAs targeting the SOCS1 CDS are blue. The SOCS1 protein domain structure is depicted above, with the SH2 and SOCS box domains labeled. NTD, N-terminal domain. sgRNAs targeting the SH2 domain of SOCS1 are depicted in light blue. (**C**) Editing efficiency of top sgRNAs identified in **B** was assessed by electroporation of Cas9/sgRNA RNPs into activated primary human T cells in an arrayed format, with editing efficiency of the cut site quantified by Amp-Seq. sgRNAs are labeled along the *x* axis, with dotted line depicting the threshold of SOCS1 sgRNAs achieving the targeted IL-2–mediated increase in pSTAT5. (**D**) Activated human primary T cells were edited with Cas9/sgRNA RNPs targeting either SOCS1 or olfactory genes in an arrayed format, with edited T cells stimulated with IL-2 and pSTAT5 signals quantified by FACS and depicted as fold change over sgOlf control. sgRNAs are labeled along the *x* axis, with dotted line depicting SOCS1 sgRNAs achieving the targeted IL-2–mediated increase in pSTAT5. (**E**) A comparison between editing efficiency (*x* axis) and pSTAT induction fold-change (*y* axis) of evaluated sgRNAs, with the u728, kipc, and qd5u sgRNAs identified as the most potent sgRNAs targeting SOCS1 based on editing efficiency and functional potency.

To extend our observation that inactivation of SOCS1 enhances the antitumor function of TIL with the objective of applying these findings for therapeutic use, we next identified potent and selective sgRNAs targeting the human *SOCS1* gene. We systematically evaluated all potential sgRNAs targeting the coding sequence (CDS) of SOCS1 by first eliminating sgRNAs predicted to cut multiple sites in the genome and then performing a CRISPR screen using an sgRNA tiling library that included 134 SOCS1-targeting sgRNAs with unique genome-cutting sites. The CRISPR tiling screen approach can serve to inform the functional impact of targeting various SOCS1 protein domains with candidate sgRNAs (47). Using our observation that SOCS1 restrains accumulation of activated human T cells in the presence of IL-2 in vitro, the sgRNA tiling library was introduced into activated primary human CD3^+^ T cells by lentivirus transduction with Cas9. The sgRNA Lib^+^ T cells were then expanded in the presence of IL-2 (Figure 5A). sgRNAs targeting the kinase inhibitory region (KIR), the extended SH2 subdomain (ESS), and the SH2 domain of SOCS1 were robustly enriched in comparison with sgRNAs targeting other regions of the protein, including the NTD and SOCS box domains (Figure 5B). The KIR domain has been found to directly block the substrate-binding groove of JAKs and the SH2 domain mediating binding to JAK proteins, with these domains having a role in SOCS1 function (48). Our data indicate that sgRNAs targeting the KIR, ESS, and SH2 domain of SOCS1 provide the most potent inactivation of SOCS1 protein function.

We next quantified the editing efficiency of the top 12 of the highest ranking sgRNAs identified in the SOCS1 CRISPR tiling screen by electroporating primary human T cells with sgRNA/Cas9 RNPs in an arrayed format and determining editing efficiency by amplicon-sequencing (Amp-Seq). We found that multiple sgRNAs were able to robustly achieve greater than 80% editing efficiency across at least 4 independent donors (Figure 5C). As SOCS1 is a negative regulator of IL-2 signaling, we also evaluated the functional potency of candidate sgRNAs in a human T cell pSTAT5 assay, using IL-2 as a stimulus. Multiple sgRNAs were able to drive enhanced IL-2–mediated pSTAT5 signals in primary human T cells in comparison with unedited controls (Figure 5D), with editing efficiency strongly correlating with pSTAT5 enhancement (Figure 5E).

To identify potent SOCS1 sgRNAs with minimal off-target edits, the most potent sgRNAs were additionally interrogated for their off-target profile using GUIDE-Seq (49). This method can detect off-target double-stranded oligodeoxynucleotide (dsODN) integration events occurring at frequencies as low as 0.1% relative to on-target insertion reads. Across 6 independent donors and 4 repeat studies, we found that the u728 sgRNA targeting the 3′ end of the SH2 domain of SOCS1 displayed strong editing efficiency, with 22 potential off-target sites identified (Supplemental Figure 5A). As GUIDE-Seq is a useful tool for discovering genomic cut sites, we next verified the on- and off-target editing profile of the u728 guide at the native loci in human melanoma-derived TILs using Amp-Seq. In addition to observing over 90% on-target editing efficiency of SOCS1 in TILs, we found that only 1 of the 22 potential off-target sites identified by GUIDE-Seq was found to be statistically different between u728-edited versus unedited TILs by Amp-Seq (Supplemental Figure 5C). Importantly, this confirmed off-target site, which occurs at position chr7:133126575 in the GRCh38 assembly (Supplemental Figure 5D), is located within an intergenic region with no known function and thus does not represent a substantial genotoxicity risk. Based on its strong potency and clean off-target profile, we prioritized the use of u728 sgRNA for the inactivation of the SOCS1 gene in TILs for therapeutic use.

### CRISPR/Cas9 inactivation of the SOCS1 gene in human TIL drives enhanced functionality and pSTAT4 sensitivity to IL-12.

**Figure 6:**
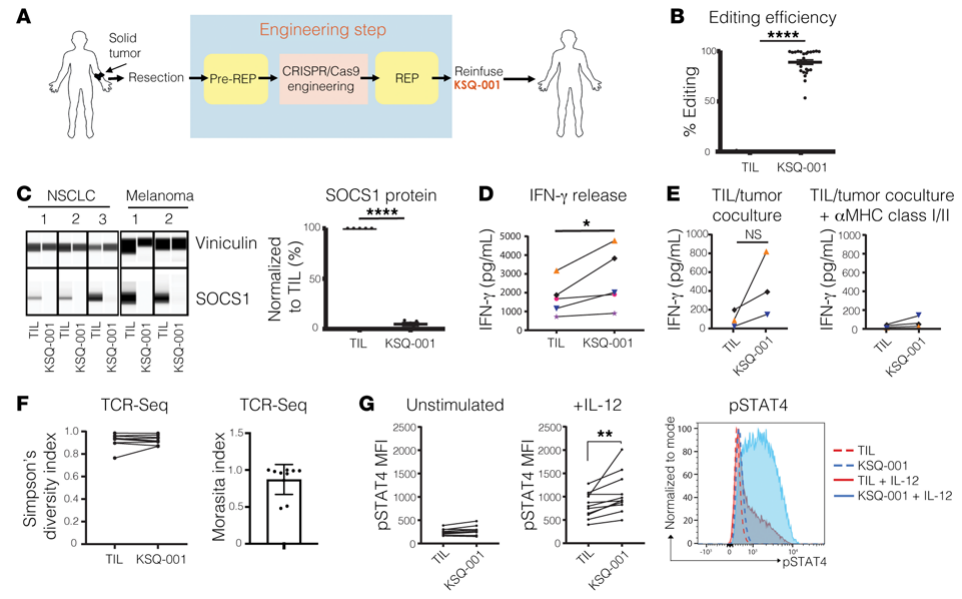
CRISPR/Cas9 inactivation of the *SOCS1* gene in human TILs drives enhanced functionality and pSTAT4 sensitivity to IL-12. (**A**) Schematic of the process for manufacturing KSQ-001, a CRISPR/Cas9-eTIL containing inactivation of SOCS1. Solid tumors are surgically resected from patients, with TIL extracted from fragmented tumor by culturing in the presence of IL-2 in a pre-REP. Following the pre-REP, u728/Cas9 protein RNPs are introduced into the TIL by electroporation, followed by expansion in a REP in the presence of irradiated PBMC feeders IL-2 and OKT3. KSQ-001 is then harvested and cryopreserved for transport to a patient for infusion (**B**) Sequencing of PCR amplicons from the u728 cut site is displayed from 15 independent donors and 26 independent data sets. (**C**) Capillary immunoassay (Wes) detection of SOCS1 protein in TIL and KSQ-001 samples from the indicated tumor type. SOCS1 protein was detected as a single band at approximately 33 kd. Vinculin (117 kd) was used as loading control. (**D**) IFN-γ release by donor-paired TILs and KSQ-001 following cryopreservation and thaw and activation with anti-CD3 tetramers. (**E**) TIL reactivity to autologous tumor digests was assessed by coculture, with IFN-γ production quantified in the absence (left) or presence (right) of anti–MHC class I and class II blocking antibodies. (**F**) Diversity and overlap of donor-paired TILs and KSQ-001 CDR3 TCR repertoire results as quantified by FR3AK-U-Seq are displayed as Simpson’s diversity index and Morisita index, respectively. (**G**) TILs or KSQ-001 was stimulated by IL-12 for 1 hour (middle) in comparison with an unstimulated control (left). pSTAT4 MFI gated on donor-paired CD3^+^ TILs and KSQ-001 is displayed. A representative pSTAT4 FACS plot from a single donor is depicted (right). For **B**–**G**, each dot represents individual donor. Statistical analyses were performed using unpaired, 2-tailed Students’ *t* test. *P < 0.05; **P < 0.01; ****P < 0.0001.

We next used the u728 sgRNA to manufacture KSQ-001, an autologous engineered TIL therapy, by inactivating the *SOCS1* gene by electroporation of sgRNA/Cas9 RNPs (Figure 6A). Across 26 independent data sets and 15 donors, we consistently observed editing efficiencies of the *SOCS1* gene of over 80% in KSQ-001 (Figure 6B), with editing of the *SOCS1* gene resulting in complete depletion of SOCS1 protein in KSQ-001 manufactured from both NSCLC and melanoma donors (Figure 6C). We observed that the electroporation-based engineering step did not affect KSQ-001 expansion during the REP (Supplemental Figure 6A), viability following cryopreservation and thaw (Supplemental Figure 6B), or activation-induced cell death (AICD) in comparison with unedited TILs (Supplemental Figure 6C). KSQ-001 consisted of CD3^+^ cells with no detectable residual tumor cells, with patient-unique frequency of CD4^+^ and CD8^+^ T cells not affected by inactivation of SOCS1 (Supplemental Figure 6D). KSQ-001 displayed a CD45RO^+^CCR7^–^ Tem phenotype following harvest, similarly to TILs (Supplemental Figure 6E), and demonstrated enhanced expression of CD25 in comparison with TILs (Supplemental Figure 6F)

IFN-γ release by TIL upon activation through the TCR is used as a surrogate measure of potency. We observed that KSQ-001 produced higher levels of IFN-γ upon TCR activation in comparison with TILs (Figure 6D). To confirm that the enhancement of IFN-γ production was driven by inactivation of SOCS1 and not an artifact of electroporation, we also observed enhanced IFN-γ production in KSQ-001 in comparison with TILs in which the *Olf1A* gene had been inactivated (Supplemental Figure 6G). We next evaluated how KSQ-001 responded to coculture with autologous tumor digests, with KSQ-001 maintaining the ability to produce IFN-γ in comparison with TILs, with cytokine release dependent upon MHC classes I and II, as inclusion of pooled antagonistic antibodies inhibited all IFN-γ production (Figure 6E). These findings demonstrate that KSQ-001 retains specificity for autologous tumor. TILs and KSQ-001 retained similar polyfunctionality for the production of IL-2, IFN-γ, and TNF-α cytokines, with no differences observed in the impact of SOCS1 inactivation in KSQ-001 across both CD8^+^ and CD4^+^ T cell subsets across multiple donors (Supplemental Figure 6H). The TCR polyclonality of TIL is an important feature thought to drive clinical efficacy by providing comprehensive coverage of all tumor antigen specificities. We therefore wanted to assess whether SOCS1 editing affected the polyclonality of KSQ-001. We found that KSQ-001 maintained high TCR diversity based on Simpson’s diversity index and showed a high degree of TCR overlap with TILs derived from the same donor based on the Morisita index values (Figure 6F), thus confirming that inactivation of *SOCS1* has no overt impact on the overall TCR repertoire of KSQ-001.

Given the role of SOCS1 as a negative regulator of cytokine signals, we evaluated the sensitivity of KSQ-001 to a panel of cytokines, including IL-2, IL-12, and IL-15, by quantifying the levels of pSTAT5 signals for IL-2 and IL-15 and pSTAT4 for IL-12. Despite inactivation of SOCS1 by the u728 sgRNA strongly enhancing pSTAT5 signals in primary human T cells in response to IL-2 (Figure 5D), KSQ-001 surprisingly did not display enhanced pSTAT5 signals in comparison with TIL in response to IL-2 (Supplemental Figure 7A) or IL-15 (Supplemental Figure 7B) when profiled directly from REP. However, KSQ-001 did display heightened responsiveness to IL-12 through increased pSTAT4 accumulation (Figure 6G), suggestive of SOCS1-dependent pharmacology. We reasoned that the disconnect between IL-2/pSTAT5 hypersensitivity observed with SOCS1-edited primary T cells, but not KSQ-001, may be driven by the long-time duration KSQ-001 spent in the presence of IL-2 and TCR activation during manufacture (~27 days), which may induce compensatory mechanisms that blunt SOCS1-driven pSTAT5 signals in response to IL-2 and IL-15, while sparing pSTAT4 responses to IL-12. In support of this hypothesis, we found that *SOCS1* edited human CD3^+^ T cells activated with anti-CD3/CD28/CD2 tetramers and IL-2 displayed enhanced pSTAT5 signals 7 days after activation (Supplemental Figure 7C), yet failed to display pSTAT5 hypersensitivity to IL-2 after 13 days of activation (Supplemental Figure 7D). These data also demonstrate that the lack of IL-2/pSTAT5 hypersensitivity observed with KSQ-001 is not driven by a fundamental difference between the biology of PBMC-derived T cells and TILs.

### Enhanced IL-2–dependent engraftment and antitumor activity by SOCS1-edited TILs following transfer into a solid-tumor model.

**Figure 7:**
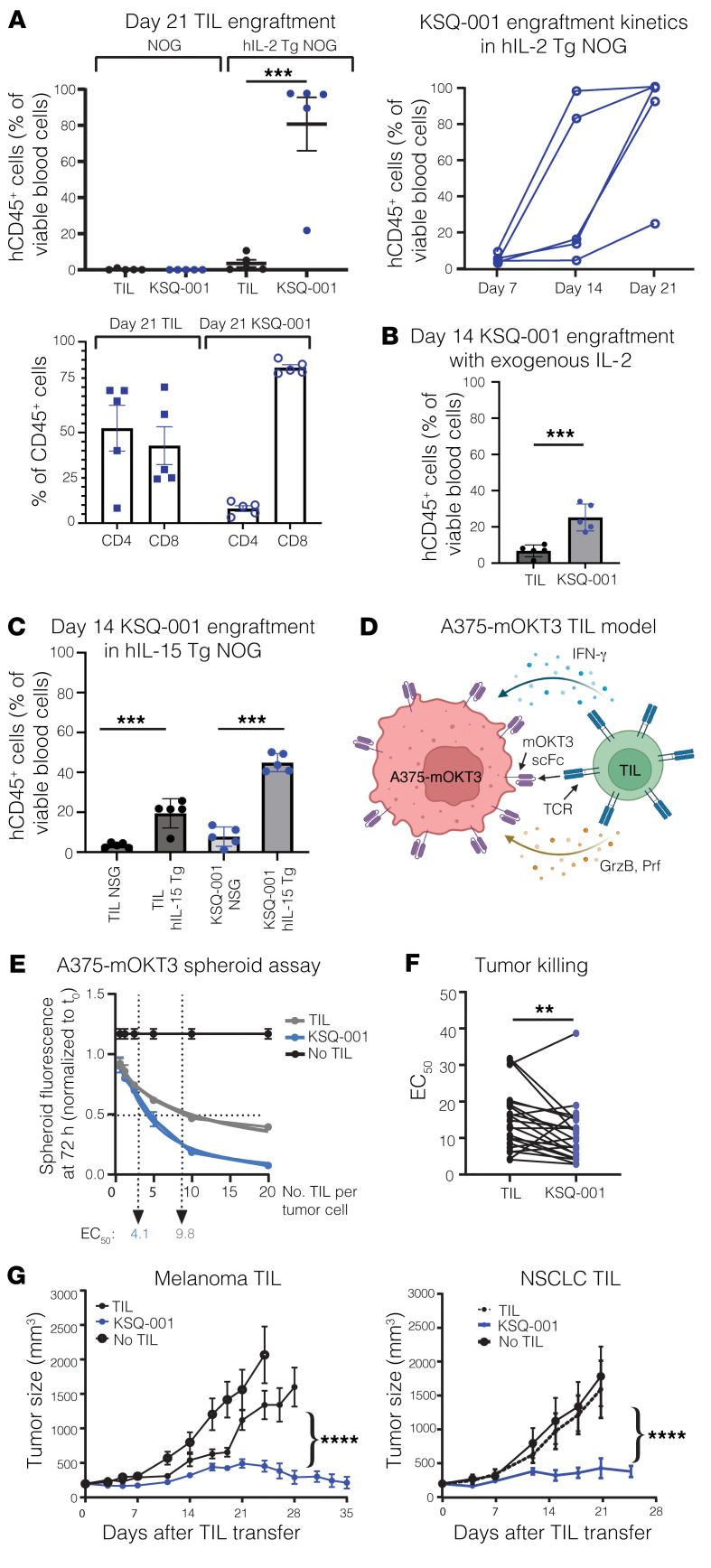
Enhanced IL-2–dependent engraftment and antitumor activity by *SOCS1*-edited TIL following transfer into a solid-tumor model. (**A**) TILs or KSQ-001 was adoptively transferred into NOG or hIL-2–Tg NOG mice, with engraftment evaluated over time by quantifying the frequency of human CD45^+^ cells present within the peripheral blood. Top row: left, frequency of human CD45^+^ cells by mouse strain and treatment group on day 21; right, frequency of KSQ-001 in hIL-2–Tg NOG mice over time. Bottom row: frequency of CD4^+^ and CD8^+^ T cells from TILs or KSQ-001 on day 21 in hIL-2–Tg mice. (**B**) TILs or KSQ-001 was adoptively transferred into NOG with 45,000 U of human IL-2 administered i.p. daily, with engraftment evaluated at day 14. (**C**) TILs or KSQ-001 was adoptively transferred into hIL-15–Tg NOG mice with 45,000 U of human IL-2 administered i.p. daily for 3 days, with engraftment evaluated at day 14 and NSG mice used as a comparator (**D**) Schematic of the mOKT3-A375/TIL model. A375 melanoma cells were engineered to express either high- or low-affinity membrane–associated OKT3 scFv binding domains that bind and agonize CD3 expressed by TILs. TILs generate an antitumor cytolytic response through production of IFN-γ and release of cytolytic granules. (**E**) TILs were cocultured with low-affinity A375-mOKT3 spheroids at various effector to target (E:T) ratios, with tumor killing assessed by Incucyte assay over time. EC_50_ kill curve at 72 hours from a representative donor is shown. (**F**) EC_50_ values from 17 donors and 22 independent paired TIL and KSQ-001 samples are shown, with each dot representing an individual paired donor. Statistical analysis was done using 2-tailed, paired *t* test. (**G**) hIL-2–Tg NOG mice bearing high-affinity A375-mOKT3 tumors approximately 100 mm^3^ in size were treated with donor-paired sgOlf-edited melanoma TILs or unengineered NSCLC TILs versus donor-paired KSQ-001, with tumor growth assessed over indicated time points. An unpaired, 2-tailed Student’s *t* test was used to evaluate statistical significance between sgOlf TIL or TIL versus KSQ-001 treatment groups in **A**–**C** and **G**, and 2-way ANOVA was used in **G**. **P < 0.01; ***P < 0.001; ****P < 0.0001.

Based on the findings above, we hypothesized that once KSQ-001 was removed from an environment replete with IL-2 and TCR activation, such as following infusion into a host, the compensatory mechanisms at play might reverse and KSQ-001 could regain hypersensitivity to IL-2. To test this hypothesis, we set out to evaluate the cytokine-dependent engraftment of KSQ-001 and TILs following adoptive transfer into immunodeficient mouse models. KSQ-001 and TILs were adoptively transferred into NOG or human IL-2 transgenic NOG mice (hIL-2–Tg NOG), with the engraftment of TILs evaluated over time. We found that both KSQ-001 and TILs failed to engraft in NOG mice, indicating there is a baseline level of cytokine support needed to support TIL and KSQ-001 engraftment not present in NOG mice. In the hIL-2–Tg NOG mouse strain, KSQ-001 displayed a marked enhancement in engraftment when compared with TILs, with KSQ-001 making up 95% of total viable blood cells in comparison with 3% for TILs, suggesting that inactivation of the *SOCS1* gene does indeed drive enhanced IL-2 signals in KSQ-001 when evaluated in an in vivo setting. Expansion of KSQ-001 in hIL-2–Tg NOG mice occurred between days 7 and 14 following transfer, with KSQ-001 presenting as primarily CD8^+^ T cells (Figure 7A). We observed donor heterogeneity in CD4 versus CD8 bias following engraftment in this experimental setting. In a clinical setting, high-dose (HD) IL-2 is administered i.v. following TIL infusion, with TILs sustained by endogenous cytokine sinks following cessation of HD IL-2 administration. As the hIL-2–Tg NOG mouse model reflects chronic exposure of TILs to HD hIL-2, we sought to evaluate the ability of KSQ-001 to engraft in 2 additional settings that may better reflect the clinical setting. We first evaluated engraftment and persistence following daily i.p. administration of 45,000 U IL-2 over 14 days, with KSQ-001 again observed to engraft and persist to a greater degree than TILs (Figure 7B). Second, we sought to model the clinical setting in which lymphodepleted patients are administered up to 3 days of HD IL-2 following TIL infusion, whereupon TILs then rely upon endogenous cytokine sinks, including IL-15, for persistence. We assessed TIL and KSQ-001 engraftment following transfer into human IL-15–Tg NOG over 14 days, with 45,000 U of exogenous IL-2 administered over the first 3 days, with NSG mice included as controls. KSQ-001 again engrafted and accumulated to a greater degree than TILs in this setting, with both KSQ-001 and TILs showing marked improvement in engraftment and persistence in the hIL-15–Tg models in comparison with NSG (Figure 7C). These data collectively demonstrate that KSQ-001 possesses hyperresponsiveness to cytokine signals in comparison with TILs following transfer in settings designed to model the clinical treatment of TILs.

A particular challenge with evaluating the antitumor activity of TILs in in vivo animal models is the patient-to-patient uniqueness and high polyclonality possessed by the TCR repertoire of TILs. We developed an MHC-independent tumor xenograft model wherein TILs manufactured across donors would recognize and generate a measurable antitumor response. We engineered the A375 human melanoma cell line with membrane-bound OKT3 (mOKT3) of both high (mOKT3) and low (mOKT3^LT^) affinity wherein polyclonal human T cells can generate a cytolytic antitumor immune response (Figure 7D). Given the high avidity of mOKT3-binding domains for the TCR, we used OKT3 engineered to possess lower functional avidity for TCR (OKT^LT^) to better recapitulate the avidity by which TCRs engage pMHC and increase assay sensitivity (50). We first developed an in vitro 3D mOKT3^LT^-A375 tumor spheroid model and performed a dose titration of KSQ-001 or TIL cocultured with mOKT^LT^-A375 spheroids and observed that KSQ-001 displayed enhanced potency in this assay in comparison with TILs (Figure 7E). This increase in KSQ-001 potency, as indicated by EC_50_ values in comparison with TILs, was observed in 19 of 22 independently evaluated donors (Figure 7F). We next used mOKT3-A375 cells in an in vivo efficacy model in the hIL-2–Tg NOG strain of mice. KSQ-001 displayed markedly enhanced antitumor activity compared with either sgOlf-edited TILs or unedited TIL from both melanoma and NSCLC donors (Figure 7G). Collectively, our data demonstrate that CRISPR/Cas9 editing of the *SOCS1* gene imparts a heightened ability of KSQ-001 to engraft and accumulate following transfer in a cytokine-dependent manner and strongly enhances the in vitro and in vivo activity in an MHC-independent solid-tumor model.

## Discussion

To rationally design a functionally enhanced T cell therapy with improved clinical activity against solid tumors, CRISPR/Cas9 screens were used to identify SOCS1 and screen for potent and selective SOCS1-targeting sgRNAs for therapeutic use, with CRISPR/Cas9 further used to inactivate SOCS1 in KSQ-001, an eTIL therapy. SOCS1 emerged as a top hit enhancing CD8^+^ T cell enrichment in tumors to a degree greater than other targets known to serve as brakes on T cell function, including CBLB, MAP4K1, and NR4A family members and PD-1. SOCS1 has previously been reported to constrain in vivo CD8^+^ T cell antitumor function (21, 51–53), in vitro human T cell proliferation (23), and in vivo CD4^+^ T cell expansion (53). Our results confirm and extend these prior studies in multiple ways. First, we demonstrate that inactivation of SOCS1 in T cell therapies can improve antitumor activity in animal models refractory to PD-1 inactivation. Second, we find that inactivation of SOCS1 markedly enhances the engraftment and accumulation of transferred CD8^+^ T cells as CD44^+^CD62L^+^ Tcm cells within the peripheral lymphoid organs, with SOCS1-edited T cells durably persisting. Importantly, we report that SOCS1 serves as a key checkpoint in the differentiation of intratumoral CD8^+^ T cells from Tex^prog^ to Tex^int^ and Tex^eff^ cells, with strong accumulation of Tex^int^ and Tex^eff^ cell subsets observed and with inactivation of SOCS1 mechanistically differing from PD-1 on the Tex cell state. These results further our understanding of the key signals affecting the differentiation of Tex cells, as discussed below. Finally, in applying CRISPR/Cas9 to discover a cell therapy candidate for clinical use, we found that sgRNAs targeting the SH2 domain of SOCS1 are the most functionally potent for therapeutic use and that inactivation of SOCS1 in human TIL leads to enhanced hypersensitivity to IL-12 and hyperresponsiveness in vivo to IL-2 as well as increased antitumor effector function.

A recent report by Galy et al. conducted genome-wide CRISPR screens to identify SOCS1 as a key checkpoint of CD4^+^ T cell accumulation and differentiation into polyfunctional Th1 cells, including boosting the antitumor function of human CAR-T cells containing a mixed population of CD4^+^ and CD8^+^ T cells (53). We report concordance between Galy et al.’s description of SOCS1-edited CD8^+^ T cells with our own observations, as both studies observed enhanced antitumor activity and accumulation as well as enhanced expression of granzyme B protein or *Gzmb* transcripts by SOCS1-edited OT1s. As our work focused on the discovery and furthering of the mechanistic underpinnings of SOCS1 in CD8^+^ T cells, our report in the context of Galy et al. provides a more comprehensive understanding of how inactivation of SOCS1 may collectively enhance the antitumor activity of a T cell drug product containing a heterogeneous mixture of CD4^+^ and CD8^+^ T cells (53). Given that SOCS1 inactivation enhanced the cytokine polyfunctionality of CD4^+^ T cells with tumors and as we identified SOCS1 as controlling the transition of Tex^prog^ to Tex^int^ and Tex^eff^ cells in the absence of provided SOCS1-inactivated CD4^+^ T cell help, we predict that the addition of SOCS1-inactivated CD4^+^ T cells and the enhanced cytokines produced within tumors will serve to further augment the impact of SOCS1 inactivation on the CD8^+^ Tex state and, by extension, antitumor activity.

A key finding in this study is our observation that SOCS1 serves as a key checkpoint in the transition of Slamf^+^CD39^–^PD-1^med^ Tex^prog^ cells to Slamf6^–^CD39^+^PD-1^hi^ Tex^int^ and Tex^eff^ cells. While not overtly changing the transcriptional state of Tex subsets, as was observed with inactivation of PD-1, the role of SOCS1 as a key brake of cytokine signals implicates cytokines as important mediators of the Tex^prog^ to Tex^int^ and Tex^eff^ transition. The role of SOCS1 at this Tex differentiation interface may be a crucial mechanism to leverage, together with enhancement of engraftment and formation of Tcm cells in the peripheral lymphoid organs, toward broadly enhancing the antitumor activity of T cell therapies. In benchmarking our studies of SOCS1 in syngeneic T cell therapy models to PD-1, we found that, while inactivation of PD-1 in transferred CD8^+^ T cells also led to increased Tex^int^ and Tex^eff^ cells, albeit to a lesser degree then SOCS1, a mechanistically distinct transcriptional state emerged that was marked by heightened expression of *Tox* and enrichment of a terminally exhausted gene signature in comparison with SOCS1 inactivation. PD-1 is known to stabilize the Tex^prog^ pool and repress the formation of CD8 subsets, with inactivation leading to erosion of the Tex^prog^ population over time (36). Our data support that genetic inactivation of PD-1 in T cells, while enhancing acute antitumor function, also accelerates the terminal differentiation of Tex cells. Corroboration of this finding includes recent reports in a murine T cell chronic exhaustion virus model as well as a clinical study evaluating the persistence of a CRISPR-engineered TCR-Tg T cell–targeting NY-ESO1 that demonstrated that genetic inactivation of PD-1 in transferred T cells was deleterious for the long-term persistence of cells in the host following transfer (54, 55). This contrasts with SOCS1, where inactivation has little impact on the Tex^prog^ transcriptional signature and with no evidence of *Tox* transcript induction observed.

We described herein KSQ-001, a CRISPR/Cas-engineered TIL with inactivation of the SOCS1 gene, with discovery facilitated using CRISPR/Cas9 tiling screens to identify sgRNAs for therapeutic use. Notably, KSQ-001 displayed markedly enhanced antitumor activity in an MHC-independent mOKT3-A375 in an in vitro spheroid and in vivo tumor model when compared with TILs. TILs are administered to patients following a course of cyclophosphamide and fludarabine-mediated nonmyeloablative lymphodepletion (NMA-LD), with patients treated with HD IL-2 immediately following infusion of TILs. NMA-LD is thought to improve the engraftment of TILs by, in part, reducing the number of competing lymphocytes present within the patient and elevating the circulating levels of available cytokines. The role of SOCS1 as an inhibitor of cytokine signals led us to explore KSQ-001 sensitivity to cytokines. Surprisingly, pSTAT5 signals to IL-2 and IL-15 by KSQ-001 evaluated directly from an REP remained similar to those of TIL. However, and relevant for how KSQ-001 may engraft in patients, KSQ-001 displayed a marked enhancement in IL-2 and IL-2 plus IL-15 cytokine-dependent engraftment and expansion in comparison with TIL following transfer into murine hosts. The similar sensitivity of KSQ-001 to IL-2 and IL-15 pSTAT5 signals to TIL when evaluated directly from a REP may be driven by the induction of other negative regulators of cytokine signaling, including other SOCS1 family members. The upstream and downstream mechanisms of SOCS1 inhibition in KSQ-001 remains an area of future inquiry. As the inactivation of SOCS1 in KSQ-001 achieves a mechanistically similar goal sought using NMA-LD and HD IL-2 in the clinic, and given the toxicities driven by NMA-LD and IL-2, KSQ-001 may enable exploring lowering or eliminating NMA-LD and IL-2 in a clinical setting. Supporting this hypothesis is the strong engraftment and long-term persistence of transferred SOCS1-edited T cells in our syngeneic studies conducted in the absence of lymphodepletion or exogenous IL-2. Consistent with our identification of cytokine-signaling pathways as playing a dominant role in the function of TILs through SOCS1, cytokine-inducible SH2-containing protein (CISH), which is a close family member of SOCS1, has also recently been described as enhancing T cell function upon inactivation (56).

In this study, we describe a systematic approach to using CRISPR/Cas9 in developing an engineered TIL product, KSQ-001, that displays markedly increased antitumor activity. Furthermore, we advance our understanding of CD8^+^ T cell biology in the context of T cell differentiation and exhaustion and implicate SOCS1 as a critical checkpoint toward the enhancement of T cell therapies for clinical use.

## Methods

### Mice.

Six- to nine-week-old female C57BL/6J, OT1 (C57BL/6-Tg[TcraTcrb]1100Mjb/J), and pmel (B6.Cg-Thy1^a^/Cy-Tg[TcraTcrb]8Rest/J) mice were purchased from The Jackson Laboratory. hIL-2 NOG (NOD.Cg-*Prkdc^scid^* IL-2rg^tm1Sug^Tg[CMV-IL-2]4-2Jic/JicTac) mice were obtained from Taconic Biosciences. To generate Cas9-Tg × TCR-Tg strains for in vivo CRISPR screens, female Cas9 mice were purchased and crossed with OT1 or pmel mice at The Jackson Laboratory. Mice were allowed to acclimatize for 1 week and were injected with tumors at between 8 and 12 weeks of age. Mice were maintained in a pathogen-free facility at the Charles River Accelerator and Development Lab (CRADL) (Cambridge, Massachusetts, USA).

### Cell lines.

For mouse syngeneic tumor models, the B16-OVA cell line was provided by Randolph Noelle (Dartmouth Medical School, Hanover, New Hampshire, USA), and the MC38-gp100 colon cell line was provided by Patrick Hwu (H. Lee Moffitt Cancer Center, Tampa, Florida, USA). The A375 human melanoma cell line was obtained from ATCC and engineered to express either a low-affinity or high-affinity membrane-associated anti-CD3 binding domain from clone OKT3 (mOKT3) together with RFP. See Supplemental Methods for more information on A375-mOKT3 cell lines.

### Cell culture.

Descriptions of cell culture of syngeneic tumor cells; generation of sgOlf, sgPD-1, and sgSocs1 OT1 and pmels; generation of human TIL; and functional assays, including IFN-γ release, AICD, TIL/tumor coculture, pSTAT, and A375-mOKT3 spheroid-killing assays, are included in the Supplemental Methods.

### CRISPR screens.

Detailed methods for the human in vitro TIL expansion screen, in vivo Cas9-Tg × TCR-Tg T cell syngeneic tumor screens, and SOCS1 sgRNA tiling screen are included in the Supplemental Methods.

### In vivo tumor models.

For the OT1/B16-OVA model, female C56BL/6J mice were inoculated with 0.5 × 10^6^ B16-OVA cells s.c. in the right flank. After 8 days for small tumor studies (~100 mm^3^, range of 80 mm^3^ to 120 mm^3^) or 14 days for large tumor studies (~300 mm^3^, range of 135 mm^3^ to 550 mm^3^) mice were randomized by tumor volume and 3 × 10^6^ sgOlf, sgPD-1, or sgSocs1 OT-1 T cells were transferred in 0.2 mL PBS via the lateral tail vein. Prior to transfer into mice, OT-1s were tested via PCR for a comprehensive list of mouse pathogens by Charles River Laboratory Testing Management. Tumor volumes were measured twice weekly using a digital caliper and tumor volume (mm^3^) calculated using the following formula: (width^2^ × length)/2, where length was the longer dimension. To assess tumor growth following rechallenge depicted in Figure 2, mice undergoing complete tumor rejection were rechallenged with 0.5 × 10^6^ B16-OVA cells s.c. on the left flank on day 61 and monitored for tumor growth. For the pmel/MC38-gp100 model: female C56BL/6J mice were inoculated with 1 × 10^6^ MC38-gp100 cells. At the indicated tumor size, mice were randomized and received 7 × 10^6^ sgOlf, sgPD-1, or sgSocs1 pmel T cells in 0.2 mL PBS i.v. via the lateral tail vein. For the NOG/hIL-2 NOG engraftment model, female NOG or hIL-2 NOG mice were dosed with 10 × 10^6^ TIL or KSQ-001 TIL i.v. in 100 ml volume. For the TIL/A375-mOKT3 model, female hIL-2 NOG mice were inoculated with 5 × 10^6^ A375-mOKT3 cells s.c. in a 50:50 mix with Matrigel (Corning Biosciences). Seven days after inoculation, 10 × 10^6^ TIL or KSQ-001 were transferred i.v.

### Tissue processing.

Single-cell suspensions were generated from spleen, lymph nodes, blood, and tumors. Spleens were processed using nonenzymatic digestion on GentleMacs protocol M spleen 01 01. Splenocytes were then RBC depleted with ACK (Lonza), filtered, washed, and resuspended in FACs buffer for staining. Lymph nodes were processed by gently pushing the tissue through a 35 μm filter, washing, and resuspending in FACs buffer for staining. Blood samples were collected by either cardiac puncture at termination or via tail vein at the indicated time points and were processed using ACK (Lonza) to deplete red blood cells. Tumor tissues were dissociated using the Mouse/Human Tumor Dissociation Kit (Miltenyi Biotec), wherein tumors were chopped into smaller pieces, incubated for 5 to 10 minutes at 37°C in the enzyme mixture, and then fully dissociated using tumor program 37C_TDK_1 as recommended by the manufacturer (Miltenyi Biotec).

### FACS analyses.

Methods for FACS analyses, including sample preparation and a comprehensive list of antibodies used, are included in the Supplemental Methods.

### Statistics.

Data from mouse efficacy studies are represented as mean ± SEM. Statistical significance between groups was determined by either 2-way ANOVA or a 2-tailed unpaired Student’s *t* test, as indicated. Statistical analyses were performed using GraphPad Prism software, version 10**.** The Simpson diversity index was used to evaluate the diversity of TCRs within a population of TILs by measuring the probability that 2 TCRs taken randomly from a population of TCRs will be different TCR sequences, with the higher the Simpson diversity index value, the greater TCR diversity in a population of TILs. The Morisita index was used to evaluate TCR overlap between 2 populations of TILs by quantifying how similar 2 TCR repertoire samples, as determined by TCR-Seq, were in terms of the overlapping TCR sequences and how well represented those sequences were within the 2 populations. Statistical significance between treatment groups in scRNA-Seq clusters depicted in Figure 4J was determined by hypergeometric test.

### Study approval.

All procedures involving the care and use of animals were reviewed and approved by the Institutional Animal Care and Use Committee (IACUC) and were conducted at the AAALAC-accredited CRADL under protocol 2021-1252 in accordance with associated regulations and guidelines.

### Data availability.

The T cell CRISPR screening data from Figure 1 and scRNA-Seq data from Figure 3 are available in Supplemental Tables 1–3, with data also uploaded and available at the NCBI’s Gene Expression Omnibus database (GEO GSE237695). Values for data points described in Figure 2, Figure 3, Figure 6, and Figure 7 as well as Supplemental Figures 1, 2, 3, 6, and 7 are reported in the Supporting Data Values file.

## Author contributions

MRS, KW, FAS, FS, LC, and MJB conceived of the experiments. CW, SS, SL, CC, ZRC, TX, HSG, LJW, FT, PRD, RAL, TEG, NC, AFH, NJT, CPB, ILM, KS, JJM, SK, and GVK conducted experiments and analyzed data. MJB wrote the manuscript. MRS, SS, CD, FS, LC, and MJB provided supervision. Co–first authorship was determined as MRS, CW, ZRC, SS, and SL served as scientific leads for functional genomics, in vivo pharmacology, computation biology and in vitro pharmacology functions, respectively, with author order determined by overall merit of study contributions.

## Supplementary Material

Supplemental data

Supplemental table 1

Supplemental table 2

Supplemental table 3

Supporting data values

## Version 1 12/15/2023

Electronic publication
