# Supplementary material for: Rational design of a SOCS1-edited tumor-infiltrating lymphocyte therapy using CRISPR/Cas9 screens
Source: J Clin Invest. 2023 Dec 15;133(24):e163096. doi: 10.1172/JCI163096 (PMC10721144; doi:10.1172/JCI163096)
Supplement: Supplemental data [file jci-133-163096-s128.pdf]

Supplementary Information

For

Rational design of a SOCS1-edited tumor infiltrating lymphocyte therapy using CRISPR/Cas9 screens

Michael R. Schlabach[1,#], Sharon Lin[1,#], Zachary Collester[1,#], Christopher Wrocklage[1,#], Sol Shenker[1,#], Conor Calnan[1], Tianlei Xu[1], Hugh Gannon[1], Leila Williams[1], Frank Thompson[1], Paul Dunbar[1], Robert A. LaMothe[1], Tracy E. Garrett[1], Nick Colletti[1], Anja F. Hohmann[1], Noah Tubo[1], Caroline Bullock[1], Isabelle Le Mercier[1], Katri Sofjan[1], Jason J. Merkin[1], Sean Keegan[1], Gregory V. Kryukov[1], Caroline Dugopolski[1], Frank Stegmeier[1], Karrie Wong[1], Fiona A. Sharp[1], Louise Cadzow[1], Micah J. Benson*[1]

[1]KSQ Therapeutics, 4 Maguire Road, Lexington, MA 02421

[#]Indicates equal contribution

*Corresponding author. Phone: (860) 478-4162; Email: mbenson@ksqtx.com

Contents

- **Supplementary Methods**
  - Design and generation of screening libraries
  - sgRNA library virus production
  - T cell transduction
  - Cell Culture
    - Culture of syngeneic tumor cells
    - Generation of sgOlf, sgPD-1, and sgSocs1 OT1 and PMELs
    - Human TIL
    - IFNγ release
    - AICD
    - TIL / Tumor co-culture
    - pSTAT Assay
    - A375-mOKT3 spheroid killing assay
  - CRISPR Screens
  - Assessment of engineering efficiency by amplicon sequencing and WES
  - scRNA-Seq
  - GUIDE-seq
  - Engineering of high- and low-affinity A375-mOKT3 cells
  - sgRNA sequences
  - Amplicon sequencing primers for on- and off-target editing assessment
  - FACS
  - Antibody Table

- **Supplemental Data**
  - Supplemental Data 1: Human TIL screen QC
  - Supplemental Data 2: OT1 / B16-Ova screen QC
  - Supplemental Data 3: PMEL / MC38-gp100 screen QC

- **Supplementary Figures**
  - **Figure S1:** Development of a syngeneic tumor models sensitive and insensitive to inactivation of PD-1 in adoptively transferred TCR-Tg CD8 T cells
  - **Figure S2:** Inactivation of SOCS1 enhances the in vivo anti-tumor potency of CD8 T cells and drives the accumulation of $CD44^{+}CD62L^{+}$ T_{cm} memory cells in blood
  - **Figure S3:** FACS analysis of innate cell subsets from sgOlf, sgSocs1 and sgPD-1 OT1s from the TME 7 days following transfer
  - **Figure S4:** scRNA-Seq of sgOlf, sgSocs1 and sgPD-1 OT1s from the TME 7 days following transfer
  - **Figure S5:** Identification of the genomic locations of u728 sgRNA off-target cut sites
  - **Figure S6:** Characterization of KSQ-001

- **Figure S7:** Sensitivity of KSQ-001, TIL, and sgSOCS1-edited $CD3^+$ T cells to IL-2 and IL-15

Supplemental Methods

Design and generation of screening libraries:
Viral vectors were constructed for both human and mouse T-cell screening. Human libraries were in a pLenti6-based lentiviral backbone that contained a human U6 promoter cassette for guide cloning, as well as a puromycin resistance marker and tagRFP fluorescent marker (pKSQ017) or a Thy1.1 selectable marker (pKSQ144). For retrovirus, the vector was a self-inactivating murine stem cell virus (MSCV) backbone containing the same human U6 promoter guide cloning cassette as well as a human CD2 marker for cell surface expression and bead enrichment of infected cells. The vectors used for TCR-Tg CRISPR screens additionally contained a library of randomly synthesized 12-mer barcodes with the nucleotide sequence BVHDBVHDBVHD (IUPAC mixed base codes). These random barcodes (theoretical diversity of 531,441 unique sequences), when randomly paired with the guide sequence during cloning, allow the ability to independently trace millions of unique infection events in immune cells.

Libraries were generated by searching for PAM sites (NGG) in the human and mouse genome that were expected to correspond to sgRNAs that would cut the coding sequence of genes of interest. sgRNAs were chosen for the library that were predicted to cut the genome in at most one site, while not overlapping with known high prevalence SNPs and having few (<10) closely related sites in the genome that could represent likely off-target sites. Several classes of controls were included, non-cutting controls, olfactory receptor cutting controls not expected to impact biology in immune cells, lethal shredder controls that cut the genome thousands of times, and (for human only) fingerprinting controls that cut high-prevalence SNPs in human essential genes and allowed internal identification of samples by donor. The final set of filtered sgRNAs were then synthesized on a microarray (Agilent oligo library synthesis) as oligonucleotides with adapters, PCR amplified, and cloned using the type IIs restriction enzyme BbsI into the final lentiviral or retroviral vector.

sgRNA library virus production
Lentivirus production: Lentivirus was generated by lipid transfection of packaging plasmids into HEK293T cells. For a 10-layer CellStack (Corning), 300 million HEK239T cells were plated in 1 L of DMEM + 10% FBS. 24 Hours later transfection was performed using 461 ug of sgRNA library plasmid, 231 ug of packaging plasmid psPax2 (Gag-Pol), and 115 ug of pMD2.G packaging plasmid (VSV-G). Plasmid DNA was added to 37.77 ml of room temperature OptiMEM media. 2428 ul of TransIT transfection reagent (Mirus Bio) was added, mixed by vortexing, and incubated for 20 minutes before proceeding. OptiMEM+DNA+TransIT mixture was added to 1 liter of DMEM + 10% FBS. Media was aspirated from HEK293T cells and fresh media containing transfection mixture was added. 24 hours after transfection the media was replaced 1 liter of UltraCulture media (Lonza) supplemented with L-glutamine. 48 hours after transfection the supernatant was harvested from the cells, incubated for 1 hour at 37C in the presence of 50 units/ml benzonase, filtered using a 0.45 uM bottle top PES filter, and concentrated by tangential flow filtration on a spectrum labs KrosFlow mPES hollow fiber filter (100kD molecular

weight cutoff). Virus was concentrated approximately 10-50x (depending on initial volumes being processed).

Retroviral Production: Retrovirus was generated by transient transfection of Phoenix-Eco retroviral packaging cells with library plasmid pools. 5 million Phoenix-Eco cells were plated per 10 dish 24 hours before transfection in DMEM + 10% FBS. Transfection is carried out by mixing 10ug of library plasmid pool with 327 ul optiMEM media. 21 ul of Mirus TransIT-293 transfection reagent is added to this mixture, vortexed briefly, and incubated for 20 mins. Transfection mixture is then added dropwise to previously plated phoenix cells and incubated. For infection of mouse CD8 cells, media is replaced with 5ml of RPMIc (RPMI + 10% HI FBS, 20 mM HEPES, 50 uM 2-mercaptoethanol). Virus is frozen and titered for subsequent large-scale transduction.

-

T cell Transduction

TIL: To transduce TIL5746, retronectin-coated non-TC-treated 6 well plates were washed with 1ml PBS, with 200ml of pKSQ017 Lib16 or RPL10a virus added and spun at 2000g for 2hrs. $5x10^6$ TIL/well in 1ml complete XVIVO-15 media were added to each well (total volume 1.2ml) so that 200 x10e6 TIL5746 were transduced with Lib16, and 10x10e6 TIL5746 were transduced with sgRNAs targeting RPL10a. Plates were spun at 1000g for 10 minutes, and then transferred to 37C for 3 hours, followed by addition of 3ml of complete XVIVO-15 to all wells, and incubation at 37C overnight.

Mouse TCR-Tg cells: To transduce mouse CD8 TCR-Tg T cells isolated from spleens and activated with Dynabeads and 4ng/ml IL-2 on Day 0, non-TC-treated 6 well plates were coated in parallel with retronectin overnight at 4C. On Day 1, plates were washed with 1ml PBS and blocked with DPBS + 2% BSA for 30 minutes. Activated mouse TCR-Tg CD8 cells were then harvested and resuspended at a concentration of $1.5x10^6$/ml in thawed concentrated lentivirus or retrovirus supernatant with 2ng/ml IL-2 and 5mg/ml protamine sulfate. 2ml of virus + CD8 mixture is pipetted in each well of the retronectin-coated 6-well plate, with plates centrifuged at 600g for 90 mins, and then transferred to a 37C TC incubator for 5 hours. 2ml of cRPMI + 2ng/mL IL-2 was then added to each well and incubated overnight at 37C. Cells were harvested, washed, and re-suspended in cRPMI + IL2 for further expansion on the following day. The lentivirus (Thy1.1) and retroviruses (CD2) used for the OT1 and PMEL transductions, respectively, contain selectable markers for positive selection, with this selection step occurring on Day 3 following transduction. On Day 4, cells were cryopreserved for future use.

Cell Culture

B16-Ova, MC38-gp100 and A375-mOKT3 cells were cultured in DMEM (Gibco, Cat# 11885076) supplemented with 10% FBS (Gibco, Cat# 10082147) at 37°C in a 5% CO_2 atmosphere. Cells were passaged two to three times per week to ensure confluency in the flask never surpassed 80%. Tumor cells were harvested in serum-free DMEM during exponential growth phase immediately prior to inoculation.

Generation of sgOlf, sgPD-1 and sgSocs1 OT1s and PMELs: Spleens were harvested from OT1 or PMEL mice, placed in 5mL StemCell Buffer and dissociated in GentleMACS C-tubes using a GentleMACS octo dissociator. Following filtration and rinsing of cells, CD8 T cell were isolated using the EasySep™ Mouse CD8+ T Cell Isolation Kit (Stemcell, Cat# 19853) according to the manufacturer's instructions. Purified CD8 T cells were activated in the presence of 4ng/ml mouse rIL-2 with mouse CD3/CD28 Dynabeads. 48 hours later, Dynabeads were removed, sgRNA / Cas9 RNPs prepared with sgRNA at 22mM and Cas9 at 15mM in IDTE, Buffer T and electroporation enhancer, and electroporated at 1700V, 20ms, 1pulse using a Neon transfection system (LifeTechnologies). Cells were expanded in the presence of 32ng/ml mouse rIL-2 for an additional 48 hours, harvested, and either transferred directly into recipient mice or cryopreserved in 90% FBS + 10% DMSO.

Manufacture of TIL:
Dissociated Tumor Cells (DTCs) from Discovery Life Sciences were thawed and seeded into 24W Grex (Wilson Wolf) at a density of $1.5x10^6$ to $2x10^6$ /ml in Pre-REP media (RPMI 1640 (Gibco, Cat#11875) supplemented with 10% Human AB Serum (Valley Biomedical, Cat# HP1022HI), 1X Penicillin/Streptomycin Solution (Gibco, Cat#15140-122), 5µg/mL Gentamicin (Gibco, Cat#15710064), 10mM HEPES (Gibco, Cat#15630080), 1X GlutaMax (Gibco, Cat#35050061), 1mM Sodium Pyruvate (Gibco, Cat#11360070) and 3000IU/mL recombinant human IL2 (PeproTech, Cat# 200-02) and cultured at 37°C with 5% CO_2 for 15-23 days. Every 2-4 days, cells were counted, with a 50% media exchange with 3000IU/mL of IL2 and split. When cell numbers reached $40x10e^6$ pre-REP TIL, TIL were harvested for CRISPR/Cas9 engineering. Briefly, expanded TILs were counted, centrifuged at 300g for 7 minutes and resuspended with MaxCyte electroporation buffer (HyClone Cat#EPB1) according to the manufacturer protocol. Ribonucleoprotein (RNP) master mixes containing Cas9 protein (Aldevron, Cat#9212) and u728 sgRNA or a7mm OLF sgRNA were added to the cell suspension and transferred to processing assembly (MaxCyte), with the specific process assembly selected based on cell numbers. Cells were electroporated on a MaxCyte ExPERT electroporator using the "Optimization #9" program. TIL were then transferred to a recovery plate and each chamber washed with REP media (1:1 ratio of RPMI 1640 and AIMV (Gibco, Cat#12055) supplemented with 5% Human AB Serum and 6000IU/mL recombinant human IL2) twice, with each wash transferred to the recovery plate, which was then incubated at 37 °C for 20 minutes. Following electroporation and recovery, TIL is transferred into G-rex vessel (Wilson Wolf) containing REP media. To initiate the REP, allogenic irradiated PBMC from 5 donors mixed at a 1:1:1:1:1 ratio were mixed with TIL at a 100 to 1 iPBMC : TIL ratio in REP media, with 30ng/mL anti-human CD3 clone OKT3 (Biolegend, Cat# 317347) added. Between Day 1 through Day 14 of REP, a 50% media exchange and 6000IU/mL of IL2, cell count or split was performed based on media change every 2-4 days. On Day 14, cells were harvested and cryopreserved using 100% CryoStor10 (STEMCELL, Cat#07930).

IFNγ release assay: 100,000 cells per well of KSQ-001 or TIL were plated into a 96well round bottom plate (Falcon, Cat#353077) in REP media, stimulated with a dose response of anti-CD3 tetramer (1µL/mL~100µL/mL) (STEMCELL, Cat#103109) for 16hr~24hr, with supernatant IFNγ

level measured by enzyme-linked immunosorbent assay (ELISA) following manufacturer's protocol (Biolegend, Cat# 430104).

AICD assay: To evaluate the ability of TIL to undergo activation-induced cell death, KSQ-001 or TIL were stimulated with a dose response of anti-CD3 tetramer (1µL/mL to 100µL/mL) (STEMCELL, Cat#103109). Following activation, cells were harvested and centrifuged at 300G for 3min, stained for CD45 (BD Bioscience, Cat# 563204), CD4 (BD Bioscience, Cat# 560836) and CD8 (BD Bioscience, Cat# 563795), fixed, permeabilized then stained for Caspase3 (Bioscience, Cat# 550914).

TIL / Tumor digest co-culture assay: To evaluate cognate reactivity of KSQ-001 or TIL against autologous tumor cells, KSQ-001 eTIL or TIL were co-cultured with DTCs at 1:1 ratio (100,000 cells/well of eTIL or TIL with 100,000 cells/well of DTCs) for 20-24 hours, with supernatant IFNg level measured by MSD following manufacturer's protocol (MesoScale Discovery, Cat# N05049A-1). HLA-ABC blocking antibody (Biolegend, Cat# 311428) and HLA-DR, DP, DQ blocking antibody (Biolegend, Cat# 361702) were included to confirm MHC dependent IFNg release.

pSTAT assay:
Pan-CD3 T cells, TIL, or KSQ-001 cells were thawed and rested in serum-free X-VIVO15 media (Lonza, Cat# 04-418Q) overnight. Either 100ng/mL IL-2 (PeproTech, Cat# 200-02), 100ng/mL IL-15 (CellGenix, Cat#1413-050) or 30ng/mL IL-12 (R&D system, Cat# 219IL005) was used to stimulated cells for 10-30min, 10-20min or 1hr respectively, with an excess of ice-cold PBS (Gibco, Cat#14190-136) then added to the cells. Cells were then centrifuged at 500G for 5min at 4°C and washed by ice-cold PBS.

In vitro A375-mOKT3 spheroid killing assay:
A375-mOKT3 'low affinity' cells engineered to express RFP were cultured in DMEM (Gibco, Cat #11885-084) supplemented with 10% heat-inactivated FBS (Gibco, Cat#16140-071) and 1% Pen/Strep (Gibco, Cat#15140122). 72 hours prior to assay initiation, cells were harvested via TrypLE (Gibco, Cat #12604-013), with 10,000 cells per well plated in 100mL RPMI (Gibco, Cat #11875-093) supplemented 10% heat-inactivated FBS (Gibco, Cat #16140-071) and 1% Pen/Strep (Gibco, Cat#15140122) in ultra-low attachment U-bottom plates (Corning, Cat#7007). On the day of assay initiation, TIL or KSQ-001 cells were added to spheroid plate in 100mL REP media supplemented with 6000IU/mL IL-2 (Peprotech, Cat#200-02). Images were taken via Incucyte S3 (Sartorius) at 4x magnification in the red fluorescence, brightfield, and phase channels every 6 hours for 6 days to monitor spheroid growth or regression. TIL cytotoxicity against spheroids was profiled by assessing red fluorescent intensity in each well, normalized to the first time coculture time point.

CRISPR Screens
Human in vitro TIL expansion screen: The 'Lib16' sgRNA library targeting 5,137 genes with 10sgRNAs/gene and 56,408 sgRNAs total, including controls, was cloned into pKSQ017 with semi-random barcodes (see Supplementary Methods for more information on library design

and lentivirus production). Lib16 includes sgRNAs targeting genes involved in T cell function, all predicted cell surface receptors, all known immune-related genes, and all genes demonstrating expression in blood.

Dissociated melanoma tumors from donor 110005746 were obtained from Conversant Bio and seeded at $1x5^6$ cells/ml in TIL-CM (RPMI 1640 + 10% HI HS, 1X HEPES, GlutaMAX, 2-Mercaptoethanol, Pen/Strep, Gentamicin) containing 300ng/ml IL-2 in a 24 well tissue culture treated plate, and incubated at 37C. Every other day, 300ng/ml IL-2 was added to cell culture assuming consumption. On Day 5, suspension cells were harvested and re-seeded at 1.5x10e6/ml in TIL-CM combined 1:1 with XVIVO-15 and supplemented with 300ng/ml IL-2, with adherent cells observed to have disappeared from the wells. Between Days 7 through Day 22, cells were expanded in IL-2 with a 1:1 ratio of TIL-CM:XVIVO-15 media supplemented with 300ng/ml IL-2 added every other day in order to maintain cell density at 1e6 cells/ml. On Day 35, cellularity was evaluated, with composition found to be 66% $CD8^+$, and 32% $CD4^+$. TIL5746 were frozen on Day 35 in CS10 freezing medium at $1x10^8$ cells/ml. On the first day of the screen (screen Day 0), $2x10^8$ TIL were thawed, washed in 40ml XVIVO-15 media twice, and resuspended to $2x10^6$ cells/ml in XVIVO-15 media containing 600ng/ml IL-2 and 1X DNase (Stemcell, Cat# 07900). In parallel, non-tissue culture treated 6 well plates were coated with 20ug/ml retronectin in PBS and incubated overnight in preparation for transduction. On Day 1, TIL were transduced with pKSQ017 lentivirus driving expression of Lib16 under control of the human U6 promoter, as well as driving expression of tagRFP under control of a UBC promoter. Semi-random barcodes were used to track individual clones. In parallel, an aliquot of TIL5746 were separately transduced with the KSQ041 lentivirus which drives expression of a sgRNA targeting the essential gene RPL10a under control of a human U6 promoter as well as mNeonGreen under control of a UBC promoter. Transduced cells were incubated in 6 well plates at 37C in XVIVO-15 overnight. On Day 2, TIL were combined from 6 well plates, spun at 300g for 5 minutes, supernatant removed, and TIL resuspended in complete XVIVO-15 media and rested overnight. On Day 3, transduced TIL5746 were electroporated with Cas9 mRNA (Trilink #L-7206, lot# T1COL01A) using an Amaxa 4D-Nucleofector unit and pulse code CA137 with Buffer P3 according to the manufacturer's instructions. Following electroporation, 80ml of pre-warmed XVIVO-15 media was added to each well, mixed, and wells combined and washed. TIL were re-suspended to $1x10^6$ in complete XVIVO-15 media, and incubated overnight at 37C. On Day 4, $5x10^7$ transduced TIL5746 were frozen to determine the input sgRNA Library distribution. The TIL Rapid Expansion Phase (REP) was initiated on 25x10e6 transduced and edited TIL with a 1:200 ratio of TIL to irradiated PBMCs derived from 5 pooled donors, 600ng/ml IL-2 and 30ng/ml OKT3 using XVIVO-15 media in a 5L Grex. Cells were incubated at 37C. On Days 8, 11 and 15, 2.5L of media was aspirated from the Grex and replaced with 2.5L complete XVIVO-15 media, with 3mgs of IL-2 added to the 5L Grex. On Day 17, TIL were counted, with $13.2x10^9$ TIL5746 obtained, and analyzed for cellularity on a FACS, with 85.5% of the TIL $CD8^+$, and 12.9% of the TIL $CD4^+$. TIL were harvested for screen analysis by freezing three 5x10e7 cell pellets for gDNA preparations. The sgRNA distribution was compared between TIL harvested on Day 4 and Day 17.

<u>In vivo Cas9-Tg x TCR-Tg CD8 T cell syngeneic tumor screens:</u> The 'Lib30' sgRNA library targets 369 genes involved in T cell function with 10sgRNAs per gene and 3089 sgRNAs total, including controls, was cloned into pKSQ044. Lib31 sgRNA library targets 1004 predicted CSR genes with 8 sgRNAs/gene and 11,148 sgRNAs total, including controls, was also cloned into pKSQ044 (see Supplementary Methods for more information on library design and lentivirus production). Cas9-Tg x TCR-Tg OT1 or PMEL CD8 T cells were isolated from freshly harvested mouse spleens and dissociated using a GentleMACS (Miltenyi), and with CD8 T cells purified by negative selection (EasySep Mouse CD8+ T cell isolation kit). CD8s were then placed in T225 flasks at a concentration of 1M CD8s/ml and activated with CD3/CD28 Dynabeads and 2ng/ml mouse rIL-2. The following day, T cells were transduced wither either lentivirus (OT1 B16-Ova screen) or retrovirus (PMEL / MC38-gp100 screen). Dynabeads were removed the following day, with cells resuspended on cRPMI + 32ng/ml IL-2. On Day 4, transduced T cells were harvested and selected by positive selection using either Thy1.1 for OT1s or hCD2 for PMELs. $5x10^6$ Thy1.1$^+$ Cas9-Tg x OT1 CD8 T cells or $7x10^6$ hCD2$^+$ Cas9-Tg x PMEL CD8 T cells were injected into tumor-bearing mice i.v. via tail vein in 200mL PBS, with $10x10^6$ CD8$^+$ T cells saved to determine the sgRNA distribution of the input population of T cells. Group sizes were 7-8 mice per library. At the indicated time following adoptive transfer of cells, mice were euthanized and blood was harvested in EDTA tubes, and stored at -80C. Tumors, spleens, tumor draining and non-draining lymph nodes were harvested and processed further for extraction of CD8 T cells using CD8a Microbeads (Miltenyi, cat# 130-049-401) from the spleen and CD45 Microbeads (Miltenyi, cat# 130-052-301) from the tumor. Tumors were digested using the Miltenyi Tumor Dissociation Kit (Cat# 130-096-730) according to the manufacturer's instructions. Genomic DNA was isolated using the Qiamp Blood Midi and Maxi kids, according to the manufacturer's instructions.

<u>SOCS1 sgRNA Tiling Screen:</u> Primary human CD8$^+$ T cells were isolated from PBMCs, with ~$300x10^6$ CD8 T cells obtained by negative selection using a EasySep Human CD8+ T cell Isolation Kit (StemCell; Cat# 17953). T cells were activated with Immunocult Human CD3/CD28/CD2 T cell activators (Stemcell; Cat# 10990) in the presence of 10U human IL-2 in X-VIVO-15 media. On the following day, CD8 T cells were harvested, counted, and transduced with lentivirus expressing 134 sgRNAs targeting genomic positions across the full length of the SOCS1 gene as well as multi-cutter sgRNAs as depleting and Olfactory genes as neutral controls. Two days after transduction, T cells were electroporated with Cas9 mRNA (Trilink) and expanded inn 10U human IL-2 in X-VIVO-15 media for 10 additional days. Cells were then harvested; DNA was extracted and amplicons spanning the lentiviral genomic regions containing the sgRNA cassettes in the library were amplified by polymerase chain reaction (PCR) and sequenced by next-generation sequencing (NGS).

<u>CRISPR Screen Pre-processing</u>**:** Counts of sgRNA frequency were generated from FASTQ sequencing files by counting the number of occurrences of each 20nt sequence READ1. For libraries that employed a unique molecular identifier the frequency of each UMI was generated by tabulating the number of occurrences of each 12nt prefix of READ2. For libraries containing UMIs the resulting count tables were further cleaned by requiring 1) the UMI matches the mixing code used to synthesize the library 2) does not contain any ambiguous/uncalled bases 3)

is not potential index-bleed (exactly matching a more abundant guide/UMI on the same sequencing run). To minimize the impact of singleton/low frequency clones a two-component mixture model was fit to the count frequency distribution and guides present in the low abundance component were dropped.

Analysis of in vitro TIL CRISPR Screens: To identify hits from the screen we calculated the fold-enrichment of each candidate gene in an “end-point” relative to a “reference” sample. The reads from raw FASTQ files were counted, and the number reads representing each guide were tabulated. For each guide, a log-ratio between the screen end-point and the reference sample was calculated. To compute a robust gene-level enrichment score, guide level scores were aggregated by taking the median enrichment score across all guides. We assigned each guide a conservative p-value equal to the percentile of its logFC among all guides in the library. To calculate a p-value for gene enrichment individual guide-level p-values were combined using Fisher’s method. The resulting p-values were adjusted for false discovery rates by the Benjamini-Hochberg method. To standardize (Z-score) the resulting scores, logFC scores were centered and scaled by subtracting the median enrichment score and dividing by the median absolute deviation to calculate an overall effect size for each gene.

Analysis of in vivo B16-OVA and MC38-gp100 CRISPR Screens: MAGeCK-mle(Version 0.5.9.3) was used to perform CRISPR screen analysis. Genes with guide numbers fewer than 5 were removed from the analysis. The number of unique UMI for each sgRNA were summarized as input clone counts. Clone counts were organized from all in-vivo tumor samples and input samples into a count matrix. Due to the large number of zero counts in the matrix, an altered RLE normalization was performed on raw count matrix by using geometric mean of gene and size factor of one sample calculated by only non-zero counts. Normalized count matrix was used as MAGeCK-mle input. Design matrix was composed of two columns: input column with all "1" as baseline level, and tumor column with only tumor samples as "1" and the rest samples as "0". Gene level beta values from MAGeCK-mle output were used to evaluate the effect size of knocking out the corresponding gene.

Assessment of Editing Efficiency

Amplicon Sequencing (Amp-Seq): To assess on- and off-target editing efficiencies in sgRNA/Cas9 RNP edited T cells, gDNA was extracted from edited T cells using the XTRACT16+ following the manufacturer’s protocol (Autogen, cat# KX110-96). Following the extraction of gDNA, a two-step library preparation method was performed. First step PCR consisted of a multiplex PCR reaction amplifying target sites, followed by a second step PCR adding on Illumina adapters consisting of indexing to allow for multiplexed NGS. Editing efficiency was then assessed by aligning reads to the regions of interest and the fraction of indel reads was calculated to yield a cutting score.

SOCS1 protein Wes: TIL or KSQ-001 cells were stimulated by ImmunoCult Human CD3/CD28/CD2 T cell activator (STEMCELL, Cat#10970) following manufacturer’s protocol overnight in REP TIL media. Cell pellets were collected, lysed by RIPA buffer (Sigma, Cat#R0278) containing Protease and Phosphatase Inhibitor (ThermoFisher, Cat# A32961). Lysates were

centrifuged at 21,000g for 10min at 4°C; supernatant was subjected to BCA assay (Pierce, Cat# 23227) for protein quantification. SOCS1 (Cell Signaling, Cat# 68631S) and loading control, Vinculin (Cell Signaling, Cat# 13901S) were detected via Wes instrument (ProteinSimple) following manufacturer's protocol.

scRNA-Seq

Droplet-base 5' single-cell RNA sequencing (scRNA-Seq) was performed by the 10x Genomics platform and libraries were prepared by the Chromium Single Cell 5' Reagent kit according to the manufacturer's protocol (10x Genomics, CA, USA). The Cell Ranger (version 6.0.1) was used for gene expression quantification, TCR sequence assembly, and cell identification. Cell level quality control was performed using the function quickPerCellQC() from scater (version 1.18.6). Per-cell size-factor normalization was performed using the computeSumFactors() function from scran (version 1.18.7). Cell lineages were annotated automatically using clustifyr (version 1.2.0) and the Haemosphere mouse RNA-seq database(58). T cells were isolated by computational gating based on expression of Cd3d, Cd3e, and Cd3g. Seurat (version 4.1.0) was used to identify clusters and perform differential gene expression analysis. Differentially expressed genes were identified using the Wilcoxon Rank Sum test. The hypergeometric test was performed to assess treatment group enrichment in each of the identified clusters. ProjecTILs (version 2.0) was used to map our data onto scRNA-Seq data from Miller et al (45). *F*gsea (version 1.26.0) was used to perform GSEA on the CD8 expressing T cells using Tex gene signatures from Miller *et al.* and Beltra(45, 59). GSVA (version 1.48.2) was used to perform GSVA on the CD8 expressing T cells using Tex gene signatures from Miller *et al.* and Beltra *et al.* The extent of clonal expansion per cluster was quantified using the StartracDiversity() function from scRepertoire (version 1.10.0).OT1 T cells were identified by searching the TCR repertoire for cells containing both the OT1 CDR3 Tcra amino acid sequence (CAASDNYQLIW) and the OT1 CD3R Tcrb amino acid sequence (CASSRANYEQYF).

GUIDE-seq

The Guide-seq protocol was modified from Tsai et al. 2015(1) and Nagendra Palani (https://www.protocols.io/view/guide-seq-simplified-library-preparation-protocol-kxygxmwool8j/v1). The double-strand oligodeoxynucleotide (dsODN) was formed by annealing the following modified single-strand DNA:

5'-Phos-G*T*TTAATTGAGTTGTCATATGTTAATAACGGT*A*T-3' and
5'-Phos-A*T*ACCGTTATTAACATATGACAACTCAATTAA*A*C-3'.

Each oligonucleotide is phosphorylated at the 5' end and includes phosphorothioate bonds (noted by asterisks). For GUIDE-Seq, primary T cells were electroporated with RNP complexes as described above with the addition of 200pmol of the annealed dsODN. The single-tail Y adapter was formed after annealing the following DNA strands: CTACAAGAGCGGTGAGT and 5'-Phos-CTCACCGCTCTTGTAGSNNNNNNNNCTGTCTCTTATACACATCTCCGAG*C. The tail of the adapter includes a unique molecular identifier (indicated by the sequence SNNNNNNNN). Double-stranded genomic DNA was quantified by the Qubit Broad Range Assay (Cat # Q32853) and 1 µg of DNA from each sample was used for library preparation using the NEBNext Ultra II

FS DNA Library Prep kit (Cat # E6177L). Genomic DNA was fragmented to 300-700bp (37°C incubation for 10 minutes) and 5 µl of 10 µM annealed adapter was used in the subsequent adapter ligation step. Purified adapter ligated DNA was used for two separate touch-down PCR enrichment reactions of dsODN containing fragments (plus and minus strand orientations each paired with a primer binding to the Y adapter). These primers are:

Minus strand dsODN primer –
TCGTCGGCAGCGTCAGATGTGTATAAGAGACAGNNNATACCGTTATTAACATATGACAACTCAATTAAA*C,
Plus strand dsODN primer – TCGTCGGCAGCGTCAGATGTGTATAAGAGACAGNNNGTTTAATT GAGTTGTCATATGTTAATAACGGTA*T,
and Adapter primer – GTCTCGTGGGCTCGGAGATGTGTATA AGAGACA*G.

The double-strand DNA products from these reactions were quantified and 5ng from each sample's plus and minus reactions were pooled for indexing PCR using primers from the Nextera DNA CD Indexes 96 well plate (Cat # 20018708). Final PCR products were quantified and library distribution was measured by D1000 TapeStation (Cat # 5067-5582 and 5067-5583). All samples were pooled by equal mass and purified by 1.2X SPRI bead clean-up. The concentration of the final pool was quantified by Kapa Illumina Library quantification kit (Cat #KK4923). Final pools were loaded onto the NextSeq with at least 10% PhiX spike-in to ensure sequence diversity.

Engineering of high- and low- affinity A375-mOKT3 cells

The A375-mOKT3 cell line was generated by infecting A375 melanoma cells with a lentiviral vector encoding a blasticidin resistance gene and either a high affinity (parental OKT3 sequence) or low affinity version (two amino acid substitutions to reduce affinity for CD3 ~1000 fold) of the mOKT3 protein.(2) Individual clones were selected and tested for their ability to stimulate TIL in vitro.

High affinity OKT3:
MERHWIFLLLLSVTAGVHSQVQLQQSGAELARPGASVKMSCKASGYTFTRYTMHWVKQRPGQGLEWIGYINPSRGYTNYNQKFKDKATLTTDKSSSTAYMQLSSLTSEDSAVYYCARYYDDHYCLDYWGQGTTLTVSSGGGGSGGGGSGGGGSQIVLTQSPAIMSASPGEKVTMTCSASSSVSYMNWYQQKSGTSPKRWIYDTSKLASGVPAHFRGSGSGTSYSLTISGMEAEDAATYYCQQWSSNPFTFGSGTKLEINSHFVPVFLPAKPTTTPAPRPPTPAPTIASQPLSLRPEACRPAAGGAVHTRGLDFACDIYIWAPLAGTCGVLLLSLVITLYCNHRNRRRVCKCPRPVVKSGDKPSLSARYV

Low affinity OKT3^{LT}:
MERHWIFLLLLSVTAGVHSQVQLQQSGAELARPGASVKMSCKASGYTFTRYTMHWVKQRPGQGLEWIGYINPSLGTTNYNQKFKDKATLTTDKSSSTAYMQLSSLTSEDSAVYYCARYYDDHYCLDYWGQGTTLTVSSGGGGSGGGGSGGGGSQIVLTQSPAIMSASPGEKVTMTCSASSSVSYMNWYQQKSGTSPKRWIYDTSKLASGVPAHFRGSGSGTSYSLTISGMEAEDAATYYCQQWSSNPFTFGSGTKLEINSHFVPVFLPAKPTTTPAPRPPTPAPTIASQPLSLRPEACRPAAGGAVHTRGLDFACDIYIWAPLAGTCGVLLLSLVITLYCNHRNRRRVCKCPRPVVKSGDKPSLSARYV

sgRNA sequences

sgRNA	Description : Species : Target	Sequence
k1vc	sgOlf : Mouse : Olfactory receptor	GGAAGAAGTACATCTGCAAG
336b	sgPD-1 : Mouse : PD-1	CGGAGGATCTTATGCTGAAC
4u9j	sgSocs1: Mouse SOCS1	GCCGGCCGCTTCCACTTGGA
u728	Therapeutic sgRNA : Human : SOCS1	GACGCCTGCGGATTCTACTG
a7mm	Control sgRNA: Human ORA1	GCTGACCAGTAACTCCCAGG
anvh	Candidate sgRNA : Human : SOCS1	GGCCGGCCTGAAAGTGCACG
v2v0	Candidate sgRNA : Human : SOCS1	GCGGCTGCGCGCCGAGCCCG
frmu	Candidate sgRNA : Human : SOCS1	CGCACCAGGAAGGTGCCCAC
xsri	Candidate sgRNA : Human : SOCS1	GGACGCCTGCGGATTCTACT
kntc	Candidate sgRNA : Human : SOCS1	GGCTGCCATCCAGGTGAAAG
8usj	Candidate sgRNA : Human : SOCS1	GCCGGCCGCTTTCACCTGGA
erv7	Candidate sgRNA : Human : SOCS1	CTTAGCGTGAAGATGGCCTC
7bv8	Candidate sgRNA : Human : SOCS1	AGCGCGCTCCTGGACGCCTG
qd5u	Candidate sgRNA : Human : SOCS1	TGGACGCCTGCGGATTCTAC
kipc	Candidate sgRNA : Human : SOCS1	AGTGCTCCAGCAGCTCGAAG
i1d4	Candidate sgRNA : Human : SOCS1	ACGCCTGCGGATTCTACTGG
CCR5 R-30	Control sgRNA : Human : CCR5	GTGTTCATCTTTGGTTTTGT
CCR5 R-25	Control sgRNA : Human : CCR5	GTAGAGCGGAGGCAGGAGGC

Amplicon sequencing primers for on- and off-target editing assessment

sgRNA	3'-Primer	5'-Primer

k1vc	TCAATAGCCATGACAGTCAGAAG	ACAGCTACCATACCTAAGATGCT
336b	TATGATCTGGAAGCGGGCAT	CAAATGCCACCTTCACCTGC
4u9j	CTTCTTGGTGCGCGACAG	GTCACGGAGTACCGGGTTAAG
u728	CACGCACTTCCGCACATT	GAGGCCATCTTCACGCTAAG
a7mm	CAATGTGATGGGGTAAATGAACA	GATATTCCTCTCCCCTTTCATG
anvh	ATGCGAGCCAGGTTCTCG	CTTAGCGTGAAGATGGCCTC
v2v0	GAGGCCATCTTCACGCTAAG	CACGCACTTCCGCACATT
frmu	GAAGAGGCAGTCGAAGCTCT	CACGCACTTCCGCACATT
xsri	CACGCACTTCCGCACATT	GAGGCCATCTTCACGCTAAG
kntc	GTGAAGATGGCCTCGGGAC	ATGCGAGCCAGGTTCTCG
8usj	ATGCGAGCCAGGTTCTCG	CTTAGCGTGAAGATGGCCTC
erv7	GAAGAGGCAGTCGAAGCTCT	CTTAGCGTGAAGATGGCCTC
7bv8	GAGGCCATCTTCACGCTAAG	CACGCACTTCCGCACATT
qd5u	GAGGCCATCTTCACGCTAAG	CACGCACTTCCGCACATT
kipc	CTTAGCGTGAAGATGGCCTC	ATGCGAGCCAGGTTCTCG
i1d4	CACGCACTTCCGCACATT	GAGGCCATCTTCACGCTAAG
CCR5R-30	CAGGGTGGAACAAGATGGATTA	GTTGAGCAGGTAGATGTCAGTC
CCR5R-25	CAGGGTGGAACAAGATGGATTA	GTTGAGCAGGTAGATGTCAGTC
U728_ON	GTCAGATGTGTATAAGAGACAGGGCGCGCAGCCGCTCGTGCG	CGGAGATGTGTATAAGAGACAGATTCCGTTCGCACGCCGATTACCGG
OT1	GTCAGATGTGTATAAGAGACAGATTATTTACTGTCTTGCTCCAGGGCTA	CGGAGATGTGTATAAGAGACAGCATTCTCACCAGAGCCCATTAGTATGA
OT2	GTCAGATGTGTATAAGAGACAGACCCCATCTGCTCTGGCCCACA	CGGAGATGTGTATAAGAGACAGTGCCATAGAACAGACTCTAACACCAGCT

OT3	GTCAGATGTGTATAAGAGACAGAGTTTCCAGCCAGGACAGCCCCACA	CGGAGATGTGTATAAGAGACAGATCTCACTCACCCAGTGGGAAGGG
OT4	GTCAGATGTGTATAAGAGACAGCACACACACACACGCACACCCTATCATA	CGGAGATGTGTATAAGAGACAGATTGTAGCCTCATATTTGCCCCCACG
OT5	GTCAGATGTGTATAAGAGACAGCTGCTCTATTTGTTACTCACCTGGGA	CGGAGATGTGTATAAGAGACAGACACTGCCATATTCAAGCAGATTAAGTA
OT6	GTCAGATGTGTATAAGAGACAGATTGCAATCTCAAATTTCTGGCCTCAA	CGGAGATGTGTATAAGAGACAGGTGAGATCCCGTCTGTGCAAAAAATTTA
OT7	GTCAGATGTGTATAAGAGACAGGGAAGTCCGGCTCCCTCTCCACATTC	CGGAGATGTGTATAAGAGACAGGCTCATCTGGCAGCCAGGACA
OT8	GTCAGATGTGTATAAGAGACAGCCAGCATGCTTCTTTCCACCACA	CGGAGATGTGTATAAGAGACAGTGATGATGCAGCTCCTCCTCAAAGGG
OT9	GTCAGATGTGTATAAGAGACAGCATGACTAACAAGCTGTGAAAGTACTCA	CGGAGATGTGTATAAGAGACAGAAGTGCTTGGTTTAATGGGAAAAGAAAA
OT10	GTCAGATGTGTATAAGAGACAGCAACACCTTGAACTAACATTACTAGCTA	CGGAGATGTGTATAAGAGACAGGGCTGTGATATTAAAGAGTAGTCCCTAA
OT11	GTCAGATGTGTATAAGAGACAGGGCAGTCATGATCAAGTCAATCCCTTG	CGGAGATGTGTATAAGAGACAGATGGAACCTTGCTATGTTGCCTAGGG
OT12	GTCAGATGTGTATAAGAGACAGCAGCAGCTGCTTTCTGTCCTCTCTTTA	CGGAGATGTGTATAAGAGACAGAGGCTCAATGATTCAAGGCCTGCT
OT13	GTCAGATGTGTATAAGAGACAGACCACCTTGATCTTGTTGCTCTGGG	CGGAGATGTGTATAAGAGACAGTCAAGAGTCTGGAGTGAGGGCCTCATG
OT14	GTCAGATGTGTATAAGAGACAGGTAGCCAAAAGTCAGTTACTACATCTCT	CGGAGATGTGTATAAGAGACAGAGACCCAAGCTTGGAAAGTGATCTTTG
OT15	GTCAGATGTGTATAAGAGACAGTGTCTGGGTGCTCTGCCCAATGCCCTGA	CGGAGATGTGTATAAGAGACAGCCTGCTCTCCCTAACAGCAGCA
OT16	GTCAGATGTGTATAAGAGACAGTGTGGGACCAATGGCTGGGCAA	CGGAGATGTGTATAAGAGACAGAAGATCTCAGCCCACCCCTAGCACCA
OT17	GTCAGATGTGTATAAGAGACAGAGTTCTAGCTTAGAAGCTCATTGCTCT	CGGAGATGTGTATAAGAGACAGCCTGTTTATCCTGAGAGCCTCTGCTTC
OT18	GTCAGATGTGTATAAGAGACAGCCAGATACCCTTACCCTGAATACTCTGC	CGGAGATGTGTATAAGAGACAGCCCATGCTGCCCATATGGCTGTAATTA
OT19	GTCAGATGTGTATAAGAGACAGGCACTACCAACCTGAAGGTGTGGCT	CGGAGATGTGTATAAGAGACAGCCTCCCGGTGAGCCTGGTGTT
OT20	GTCAGATGTGTATAAGAGACAGCATCCTCTTCTGAGAACCAACAATTCCA	CGGAGATGTGTATAAGAGACAGCCATCGGGTCCAGCCACCA
OT21	GTCAGATGTGTATAAGAGACAGATCAGCAAGCAAGAGCCTTCCTGG	CGGAGATGTGTATAAGAGACAGGGCTGAGAGGCATTGCTGACG
OT22	GTCAGATGTGTATAAGAGACAGCGGCCTGCCTGCGACCCGAGA	CGGAGATGTGTATAAGAGACAGCTCCACTGCACCTCGCCAGA

FACS

Single cell suspensions were processed on a 96 well plate (Falcon, Cat#353077) for flow cytometry. Cells were washed with FACS buffer (Biolegend, Cat#420201) and stained with a master mix of antibodies, viability dye (Ebioscience, Cat# 65-0865-14) and Fc block (Biolegend, Cat#422302) in FACS buffer for surface staining. Following surface staining, cells were washed by FACS buffer, fixed by fixation buffer (Biolegend, Cat#420801). For pSTAT assay, following surface staining, cells were washed by FACS buffer, fixed by Cytofix buffer (BD Biosciences, Cat#554655) and permeabilized by BD Phosflow Perm Buffer III (BD Biosciences, Cat#558058). Selected pSTAT antibody was used for pSTAT staining. Samples were acquired on BD Fortessa and analyzed using Flowjo software (V10, Treestar).

FACS Antibodies

Antigen	Color	Vendor	Cat #	Antigen species	Figures
CD4	PerCP Cy5.5	BD Bioscience	561115	Mouse	2
CD8	APC	BD Bioscience	553035	Mouse	2
Va2	PE	BD Bioscience	553289	Mouse	2
CD45	BUV661	BD Bioscience	612975	Mouse	3
CD3	BV650	BD Bioscience	564378	Mouse	3
CD8	BUV395	BD Bioscience	563786	Mouse	3
CD4	BUV496	BD Bioscience	612952	Mouse	3
Vb5.1/2	BV421	BD Bioscience	742998	Mouse	2, 3
Va2	BB700	BD Bioscience	746041	Mouse	3
Slamf-6	APC	Biolegend	134610	Mouse	3
Foxp3	PE	eBio	12-5773-82	Mouse	3
CD44	BUV737	BD Bioscience	612799	Mouse	2, 3
CD39	PE.Cy7	Biolegend	143806	Mouse	3
CD62L	BV605	BD Bioscience	563252	Mouse	2, 3
PD-1	FITC	Biolegend	135214	Mouse	3
CD45	BV786	BD Bioscience	564225	Mouse	S3
CD11b	BUV737	BD Bioscience	612800	Mouse	S3
CD11c	PE	Biolegend	117308	Mouse	S3
Ly6C	FITC	Biolegend	128006	Mouse	S3
Ly6G	BB700	BD Bioscience	566435	Mouse	S3
NKp46	APC	Biolegend	137608	Mouse	S3
CD103	BV421	BD Bioscience	562771	Mouse	S3
MHC-II	BV605	BD Bioscience	563413	Mouse	S3
F4/80	BUV395	BD Bioscience	565614	Mouse	S3
CD115	BV650	BD Bioscience	750890	Mouse	S3
pSTAT5 (pY694)	PE	BD Bioscience	612567	Human	6
pSTAT4 (pY693)	PE	BD Bioscience	558249	Human	6
CCR7	Alx 647	BD Bioscience	560816	Human	S6
CD45RA	PerCP Cy5.5	BD Bioscience	563429	Human	S6
CD45RO	PE Cy7	BD Bioscience	560608	Human	S6
Live/Dead	e780	ebioscience	65-0865-14	N/A	All FACS experiments
CD45	BV510	BD Bioscience	563204	Human	S6
MCSP	Alx 647	BD Bioscience	562414	9.2.27	S6
CD3	BV421	BD Bioscience	563798	SK7	S6
CD25	BUV661	BD Bioscience	741685	Human	S6

CD4	Alx 700	BD Bioscience	560836	Human	S6E
CD8	BUV395	BD Bioscience	563795	Human	S6
IFNg	PE-Cy7	BD Bioscience	560741	Human	S6
TNFa	Alx488	BD Bioscience	557722	Human	S6
IL-2	PE	BD Bioscience	559334	Human	S6

Supplemental Data

Supplemental Data 1: Human TIL Screen QC

Background: This screen investigated the effect of CRISPR knockout on human TIL using lib16, a sub-genome library. The following table describes the two samples that were compared.

1. Results: The screen analysis was performed on zorya. A QC report was generated with clean_count output saved.

   1.1: Description of samples included in analysis:

Library	Day of Analysis	Number of Samples
Lib16	Day 4	1
Lib16	Day 17	1

2. Library/Screen QC

2.1: Screen sequencing purity: What fraction of sequenced reads match to the expected library design?

Recovered sgRNAs: 85%. Missing: 15%.

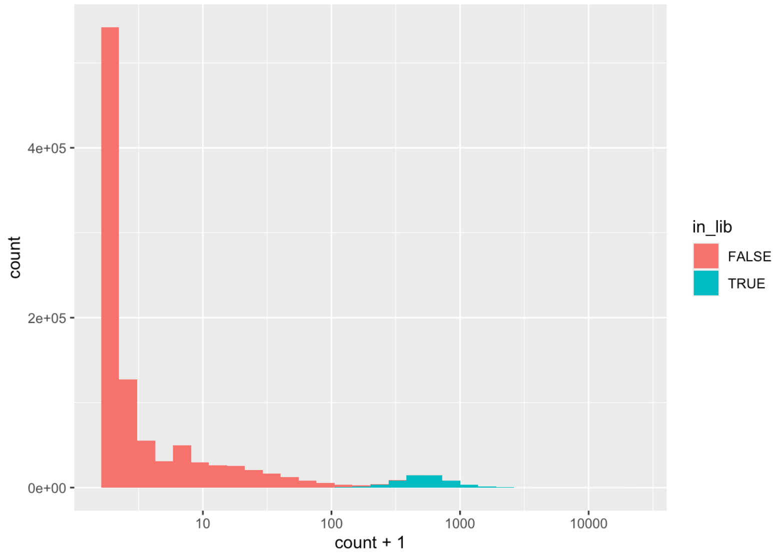

The libraries look as expected, majority of reads match to the library design, and the sequences matching the library design have much higher counts than sequences that are not part of the library design.

2.2: Screen Coverage

We expect the majority of the sgRNA library to be recovered.

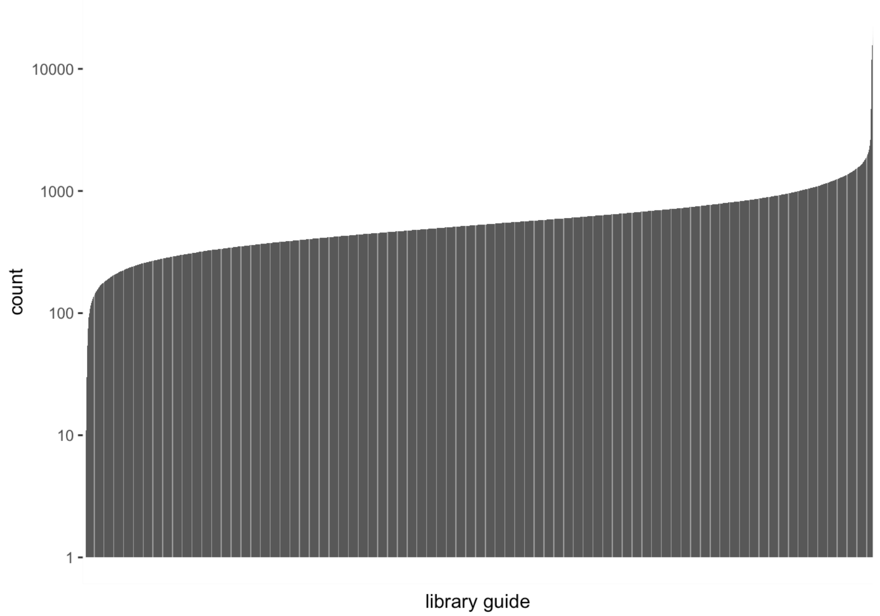

Majority of guides have uniform counts.

2.3: Gene Level QC

2.3.1: Negative controls should drop out

lib16 included controls targeting multiple positions across the genome (type="control_multicutter"). We expect these guides to drop out compared to the average guide in the library.

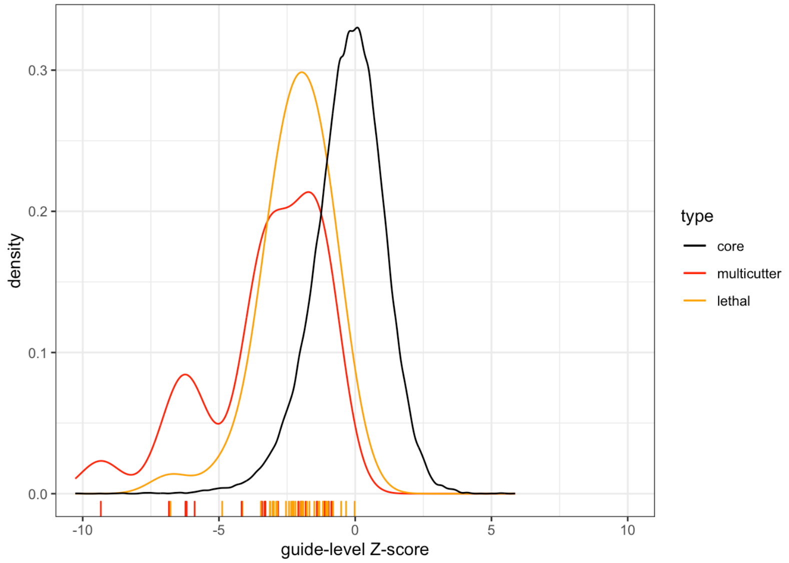

Control guides targeting essential genes or high-copy regions of the genome drop out, confirming Cas9 activity.

2.3.2: Concordance with DepMap. Since there are core fitness genes shared by all cells, we expect gene scores should roughly correlate with average DepMap dependency scores.

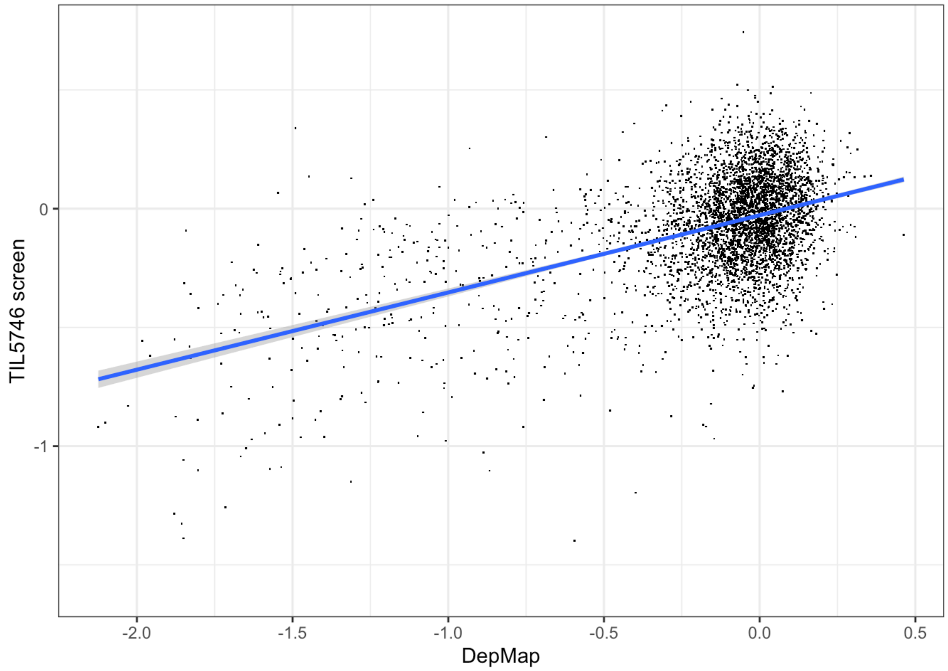

The gene scores for this screen recapitulate the pattern of core essential genes from DepMap.

Supplemental Data 2: OT1 / B16-Ova screen QC

Background: This screen investigated the effect of CRISPR knockout on OT1 T cells in vivo. Two sub-genome libraries, lib30 and lib31 were used.

3. Results: The screen analysis was performed on zorya. Both libraries (lib30 and lib31) were merged together to perform the screen analysis, with normalized clone counts and a merged sgRNA library file. A QC report was generated with clean_count output saved.

   1.1: Description of samples included in analysis:

Library	Day of Analysis	Number of Samples
Lib30	Day 14	7
Lib30	Day 21	7
Lib31	Day 14	7
Lib31	Day 21	7

4. Library/Screen QC

2.1: Screen sequencing purity: What fraction of sequenced reads match to the expected library design?

Recovered sgRNAs: 97%. Missing: 3%.

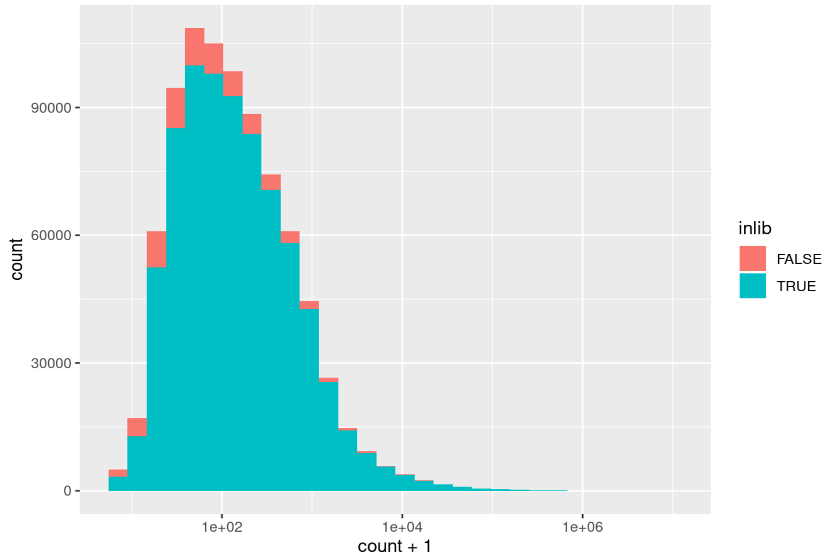

The majority of reads match to the library design, and the sequences matching the library design have much higher counts than sequences that are not part of the library design.

2.2: Screen Coverage

We expect the majority of the sgRNA library to be recovered.

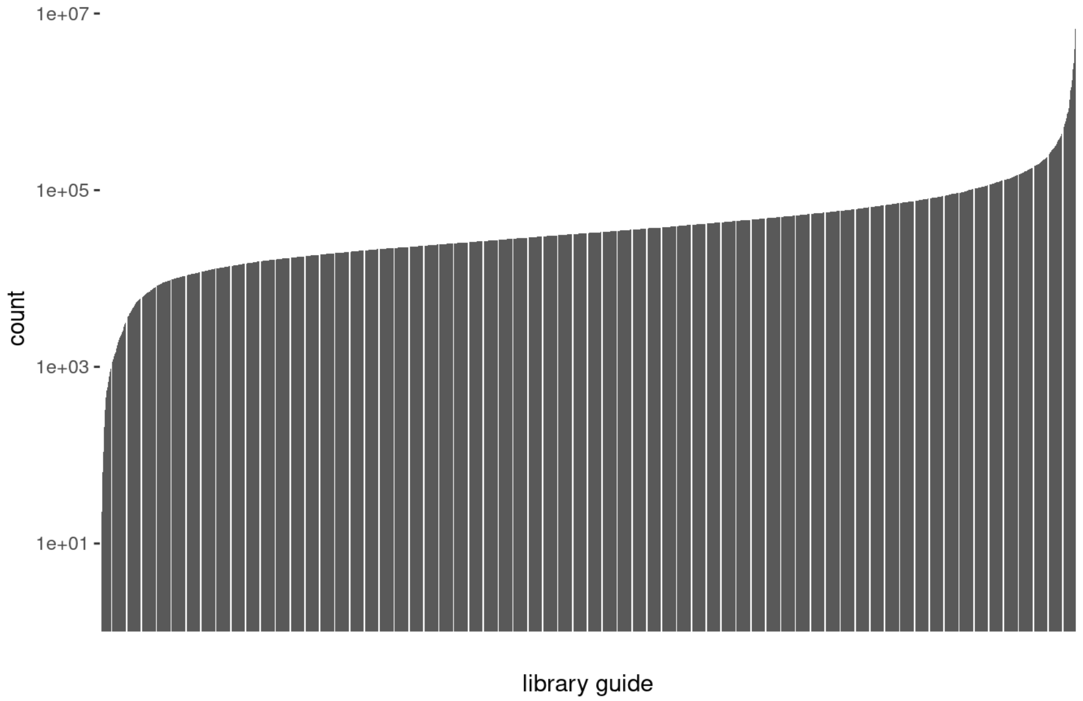

Majority of guides have uniform counts.

2.3: Gene Level QC

2.3.1: Negative controls should drop out

lib30 and lib31 included controls targeting multiple positions across the genome (type="control_multicutter"). We expect these guides to drop out compared to the average guide in the library.

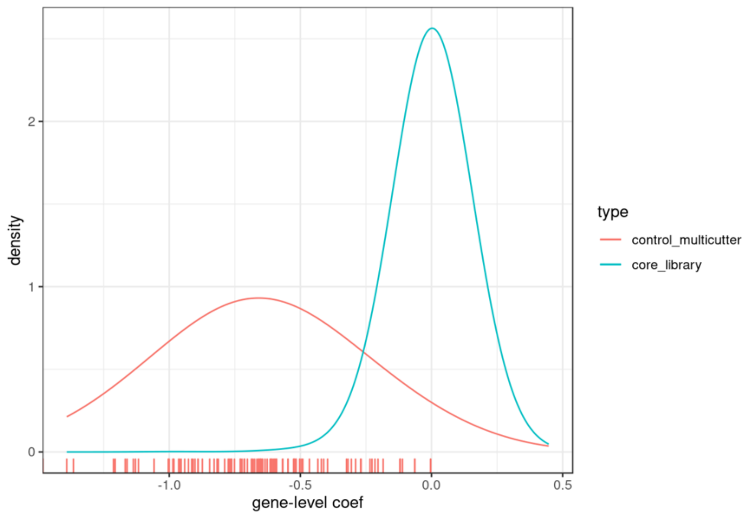

Control guides targeting high-copy regions of the genome drop out, confirming Cas9 activity.

2.3.2: Neutral controls should not enrich/deplete. Two other types of control guides were included in the library:

- **control_ORuniquecutter** are guides cutting unique locations within Olfactory receptor genes, which are not expressed in T cells.
- **control_randomnoncutting** guides don't target any sequence in the mouse reference genome and are not expected to cut. Non-cutting guides are expected to have a slight growth advantage compared to guides that create a double stranded break.

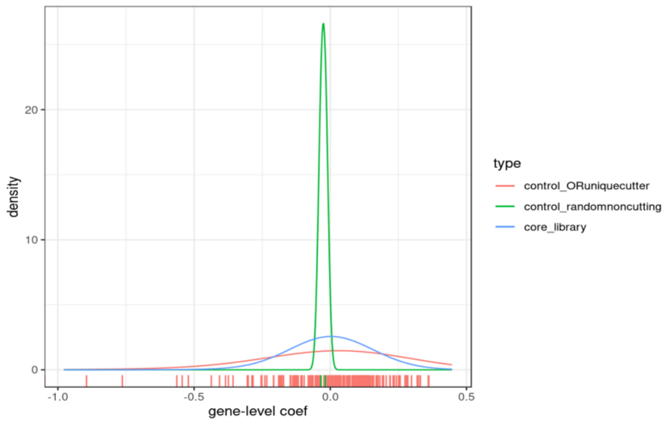

Both Olfr cutters and noncutting guides distributed nicely with coefs centered at 0, with no growth advantage observed for random-noncutting guides.

Supplemental Data 3: PMEL / MC38-gp100 screen QC

Background: This screen investigated the effect of CRISPR knockout on PMEL T cells in vivo. Two sub-genome libraries, lib30 and lib31 were used.

5. Results: The screen analysis was performed on zorya. Both libraries (lib30 and lib31) were merged together to perform the screen analysis, with normalized clone counts and a merged sgRNA library file. A QC report was generated with clean_count output saved.

   1.1: Description of samples included in analysis:

Library	Day of Analysis	Number of Samples
Lib30	Day 14	7
Lib31	Day 14	7

6. Library/Screen QC

2.1: Screen sequencing purity: What fraction of sequenced reads match to the expected library design?

   Recovered sgRNAs: 82%. Missing: 18%.

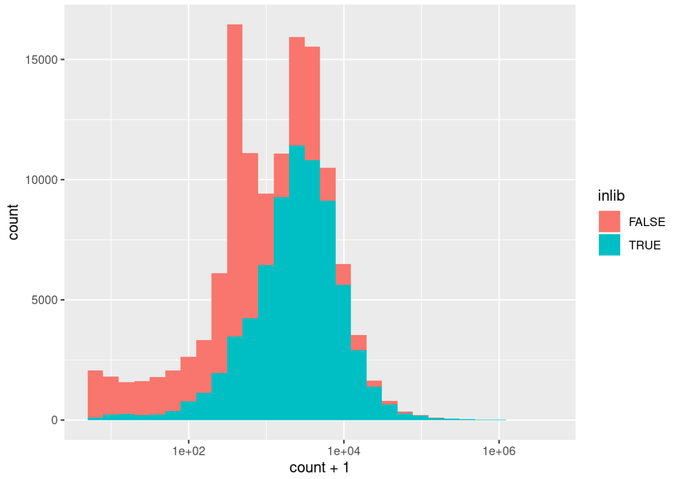

The majority of reads with high counts match to the library design. For guide sequences with counts below 1,000, majority of them are noise sequences that doesn't belong to the library sgRNA.

2.2: Screen Coverage

We expect the majority of the sgRNA library to be recovered.

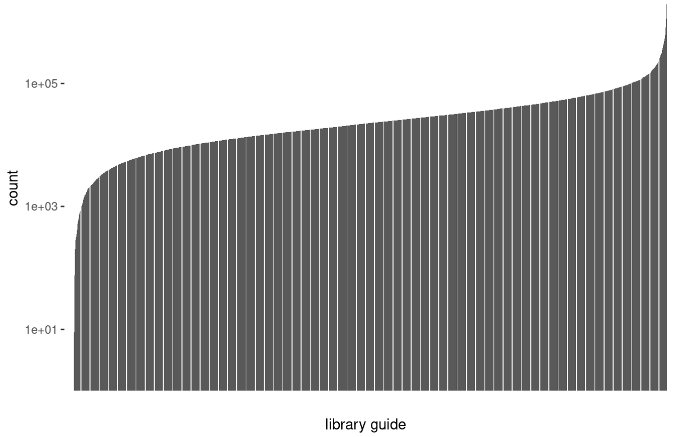

Majority of guides have uniform counts.

2.3: Gene Level QC

2.3.1: Negative controls should drop out

lib30 and lib31 included controls targeting multiple positions across the genome (type="control_multicutter"). We expect these guides to drop out compared to the average guide in the library.

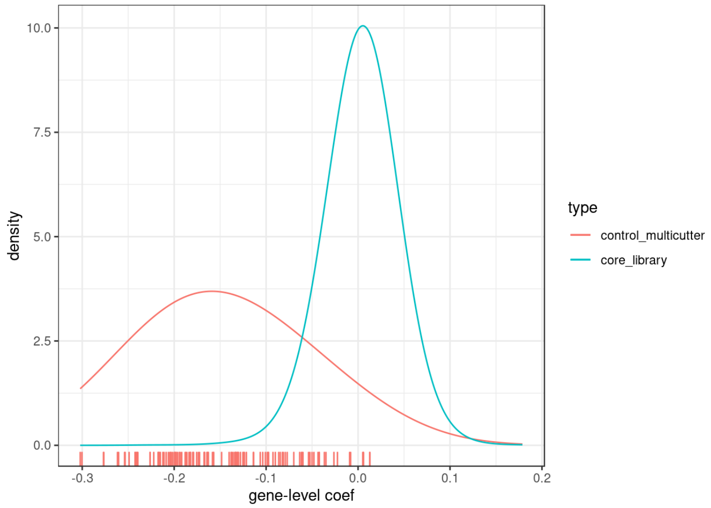

Control guides targeting high-copy regions of the genome drop out, confirming Cas9 activity.

2.3.2: Neutral controls should not enrich/deplete. Two other types of control guides were included in the library:

- **control_ORuniquecutter** are guides cutting unique locations within Olfactory receptor genes, which are not expressed in T cells.
- **control_randomnoncutting** guides won't target a specific genomic location. They should have growth advantage.

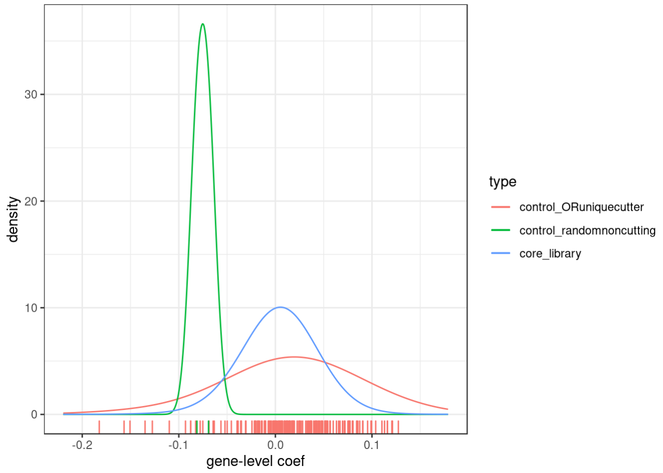

Olfr cutters distributed as expected, with coefs centered at 0. Non-cutting guides are limited in numbers; Using Olfr cutters as reference, both non-cutting guides stay within the dynamic range of neutral guides. No growth advantage observed for random-noncutting guides.

Supplemental Figures

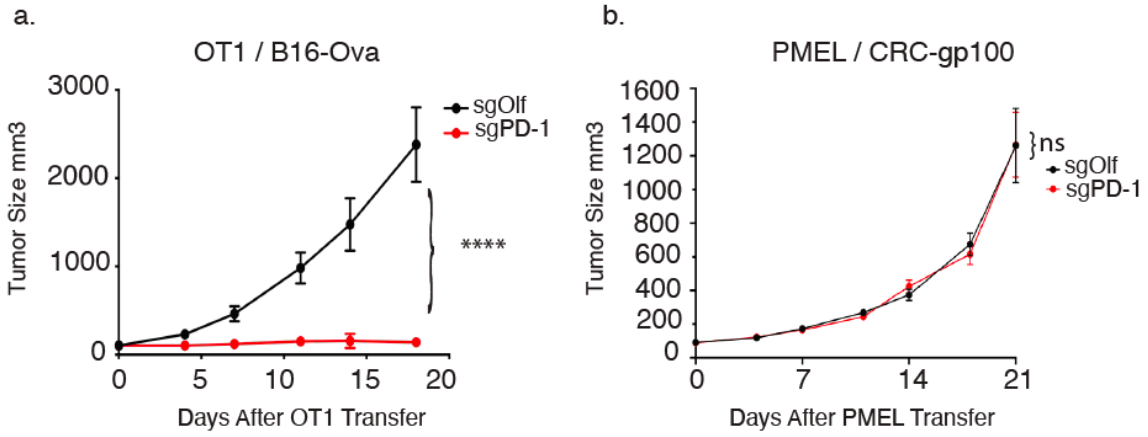

Figure S1: Development of syngeneic tumor models sensitive and insensitive to inactivation of PD-1 in adoptively transferred TCR-Tg CD8 T cells

C57BL/6 mice bearing the indicated tumor at a median size of $100mm^3$ on the flank were treated with $3x10^6$ OT1 CD8 T cells or $7x10^6$ PMEL CD8 T cells inactivated for either PD-1 (sgPD-1) or OLF1 (sgOlf) as indicated. **(a)** B16-Ova growth curves over time following transfer demonstrates that sgPD-1 OT1 T cells can control growth **(b)** MC38-gp100 growth curves over time following transfer demonstrates that sgPD-1 PMEL CD8 T cells are unable to control tumor growth. Data expressed as mean +/- SEM, with ns = no significance and **** = p value < 0.0001 by 1-way ANOVA with between the indicated comparator groups.

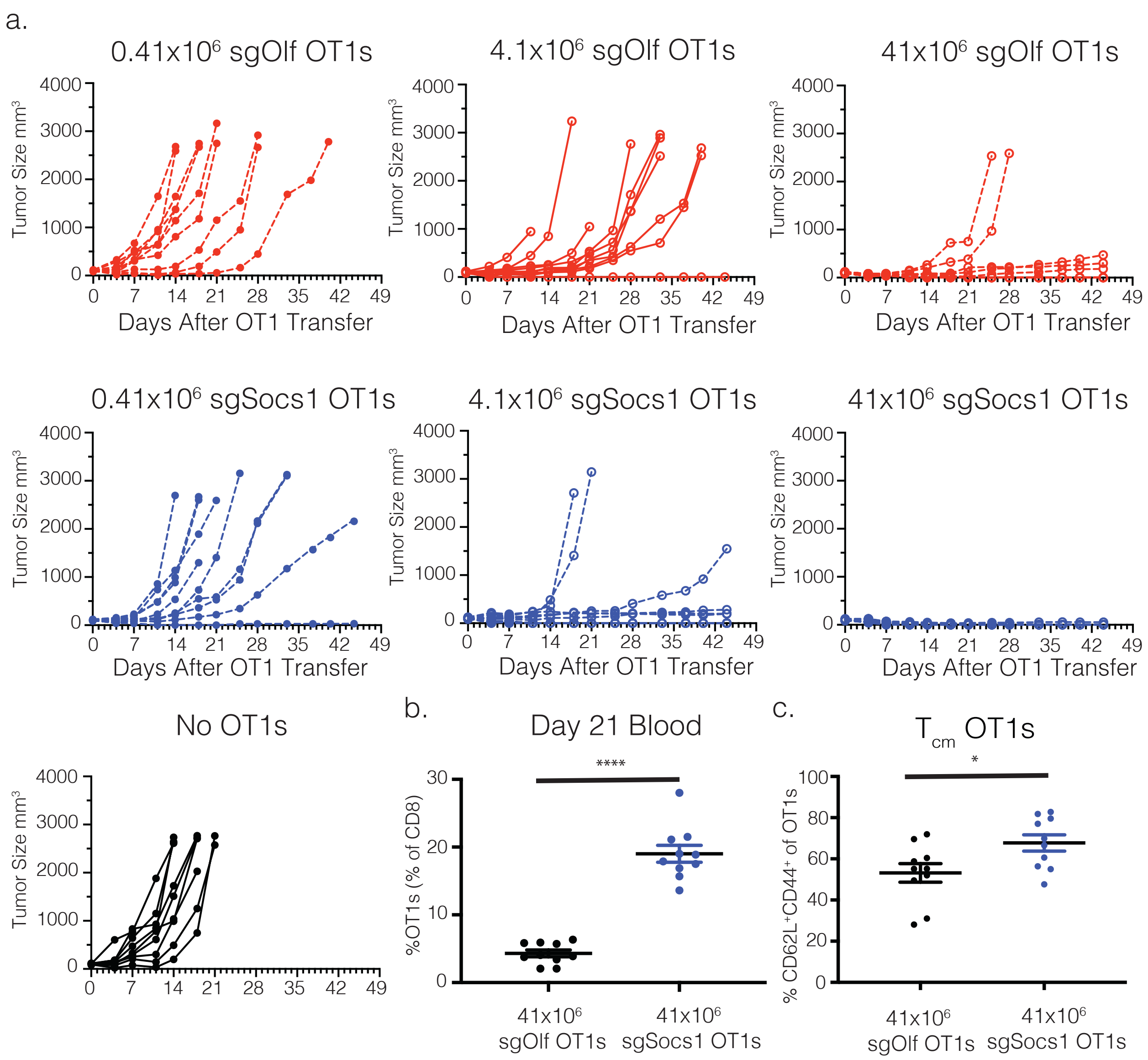

Figure S2: Inactivation of SOCS1 enhances the in vivo anti-tumor potency of CD8 T cells and drives the accumulation of $CD44^+CD62L^+$ T_{cm} memory cells in blood

C57BL/6 mice bearing B16-Ova tumors cells at a median size of 100mm^3 on the flank were treated with SOCS1 (sgSocs1) or OLF1 (sgOlf) OT1 CD8 T cells as indicated. Editing efficiencies for target genes were 91% for sgSocs1 and 71% for sgOlf. **(a)** Tumor growth curves over time are depicted. **(b)** The frequency of $Va2^+Vb5.1^+$ OT1s present in the peripheral blood of mice receiving a dose of 41x10^6 OT1s is depicted 21 days following transfer. **(c)** the frequency of $CD44^+CD62L^+$ T_{cm} and $CD44^+CD62L^-$ T_{em} OT1s were quantified in mice receiving 41x10^6 OT1s 21 days following transfer. Each dot represents an individual mouse, with **** = p value < 0.0001; and * = p value < 0.05 by unpaired, two-tailed Student's t test between the indicated comparator groups.

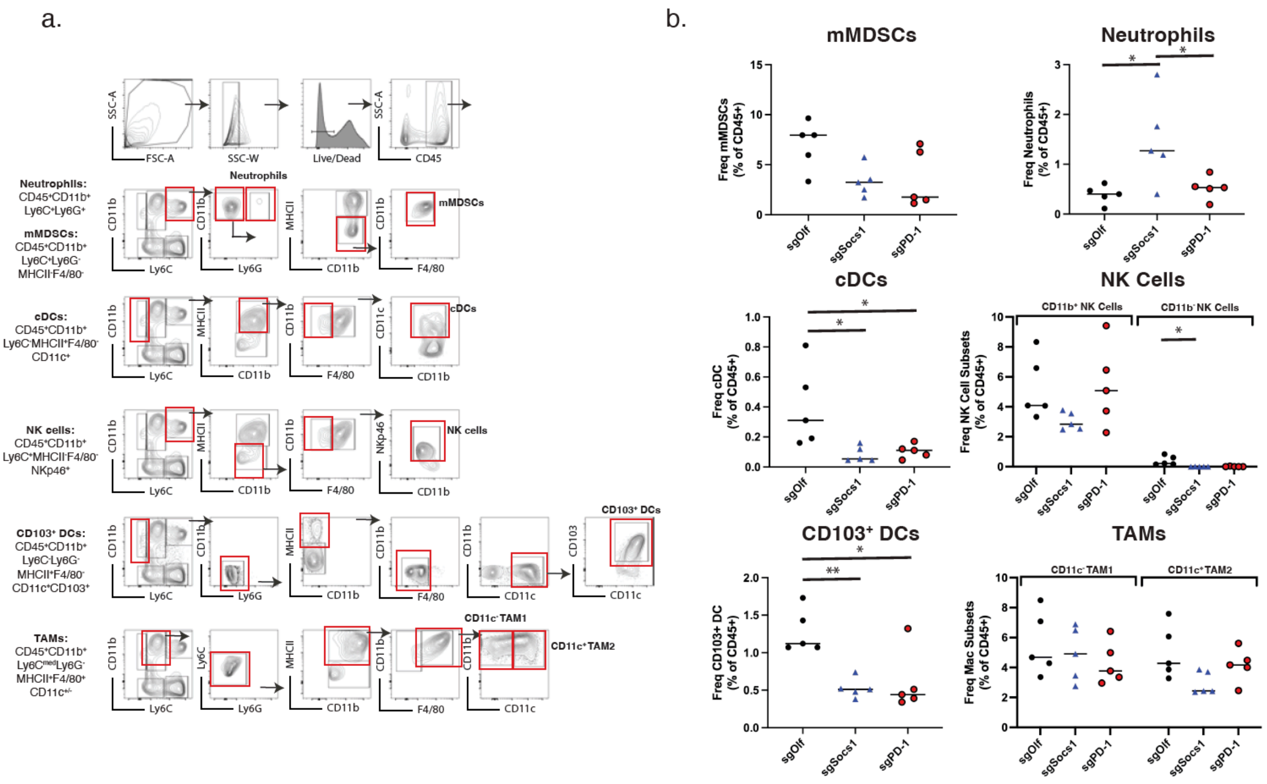

Figure S3: Inactivation of SOCS1 in transferred CD8 T cells drives increased neutrophils and reduced mMDSCs, CD103⁺ DCs and TAM2s in the TME

C57BL/6 mice bearing B16-Ova tumors cells at a median size of 100mm^3 on the flank were treated with SOCS1 (sgSocs1) or OLF1 (sgOlf) OT1 CD8 T cells as indicated. Tumors were harvested at Day 7, and FACS analyses performed to quantify innate cell populations. **(a)** FACS gating scheme is depicted to identify neutrophils, monocytic myeloid-derived suppressor cells (mMDCs), classical dendritic cells (cDCs), natural killer cells (NK cells), CD103+ dendritic cells (CD103$^+$ DCs), and type 1 and 2 tumor-associated macrophages (CD11c$^-$ TAM1, CD11c$^+$ TAM2). **(b)** The frequency of each innate cell population is depicted by treatment group, with each symbol representing an individual mouse, with *** = p value < 0.001; and * = p value < 0.05 by One-way ANOVA. p-values within each experiment were adjusted for multiple testing correction using Benjamini-Hochberg method.

a.

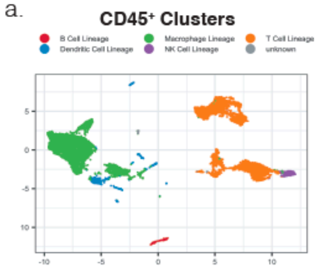

b.

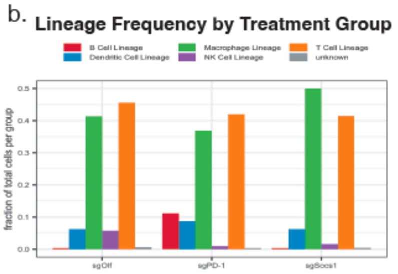

c.

T cell Cluster Total Counts

	C0	C1	C2	C3	C4	C5	C6	C7	C8	C9	C10	C11	Total
cell counts	2000	1155	1084	812	713	639	358	356	348	315	227	116	8123
% sgSocs1	627	500	360	407	260	433	14	138	90	59	51	44	
% sgPD-1	211	139	255	192	138	109	311	49	127	63	49	46	
%sgOlf	1162	516	469	213	315	97	33	169	131	315	127	26	
Totals	2000	1155	1084	812	713	639	358	356	348	315	227	116	

d.

T cell Cluster Marker Genes

C0	Tyrobp	Fcer1g	Klra7	Cd7	Pmepa1	Emb	Pde2a	Klra1	Trbv12-2	1700025G04Rik
C1	Ccl5	Gzmf	Lgals3	Trbv31	Trbv14	S100a4	S100a6	Crip1l	Rgs16	Nkg7
C2	Trbv12-1	Ccr7	2310001H17Rik	Trbc2	Hspb1	Dapl1	Fam162a	Slamf6	Dusp4	Gdi2
C3	Hist1h2ae	Hist1h1b	Ube2c	Pclaf	Top2a	Mki67	Tuba1b	Hist1h2ap	Tubb5	Stmn1
C4	Ramp3	Areg	Tnfsf11	Rora	Cd4	Cd40lg	Odc1	Gatl1	Icos	Tmem64
C5	Gm156	Gzmb	Ccl3	Ccl4	Mt1	Plac8	Spp1	Lgals3	Serpinb1a	S100a4
C6	Hspa1b	Hspa1a	Igfbp4	Fos	Klf2	Jun	Dapl1	Gm20400	Dnajb1	Lef1
C7	Xcl1	Ccl1	Pclaf	Top2a	Tuba1b	Hmgb2	Stmn1	Ube2c	Mki67	Ran
C8	Ccr7	Fam241a	Tubb2a	Rflnb	Lef1	Satb1	Gbp2	Igfbp4	Dusp10	Igtp
C9	Hspa1b	Hspa1a	Jun	Fos	Klf2	Dnajb1	Id3	Dusp1	Hspb1	Cd69
C10	Foxp3	Tnfrsf4	Ctla4	Areg	Maf	Izumo1r	Capg	Lrrc32	Il2ra	Sdf4
C11	Gm26917	Lars2	Gm42418	Ccnb1ip1	AY036118	Malat1	Cacnb3	Gm10800	Mreg	Gm49774

e.

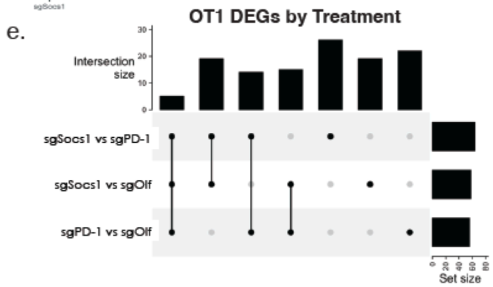

f.

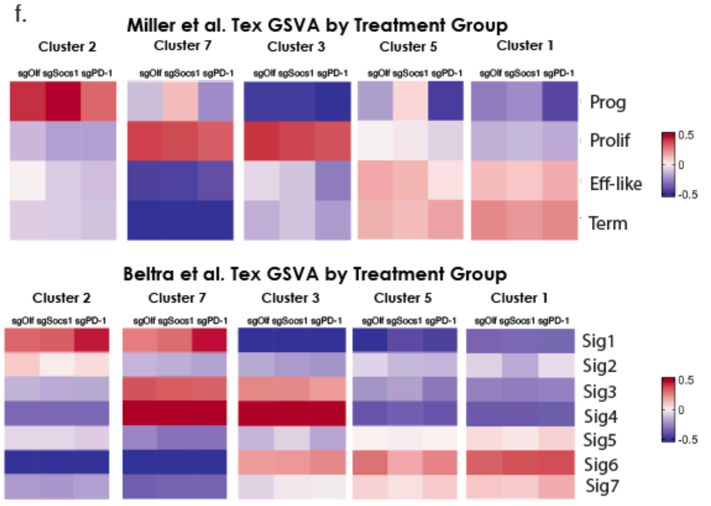

g.

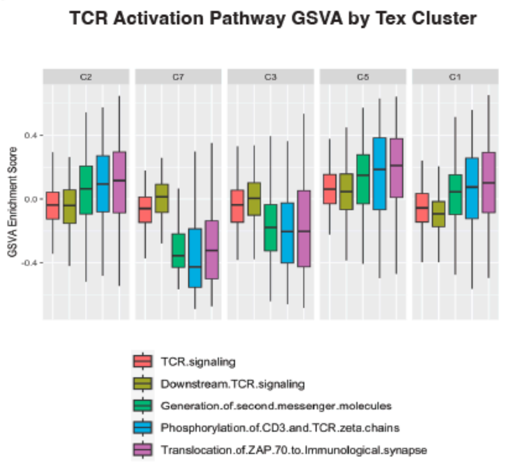

h.

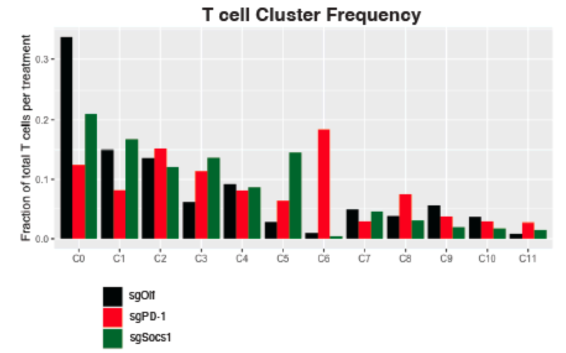

i.

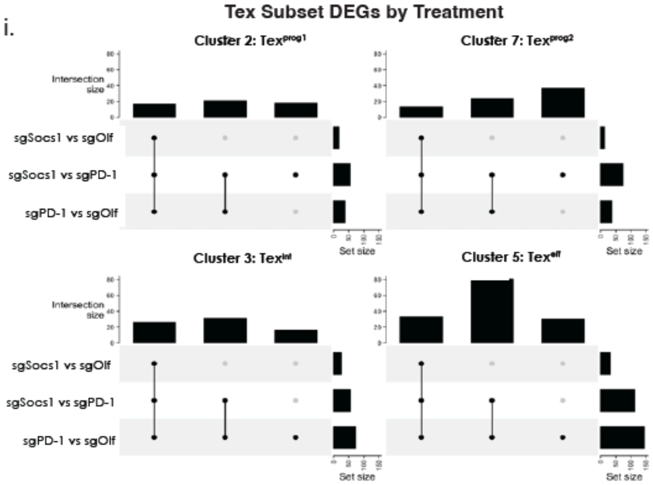

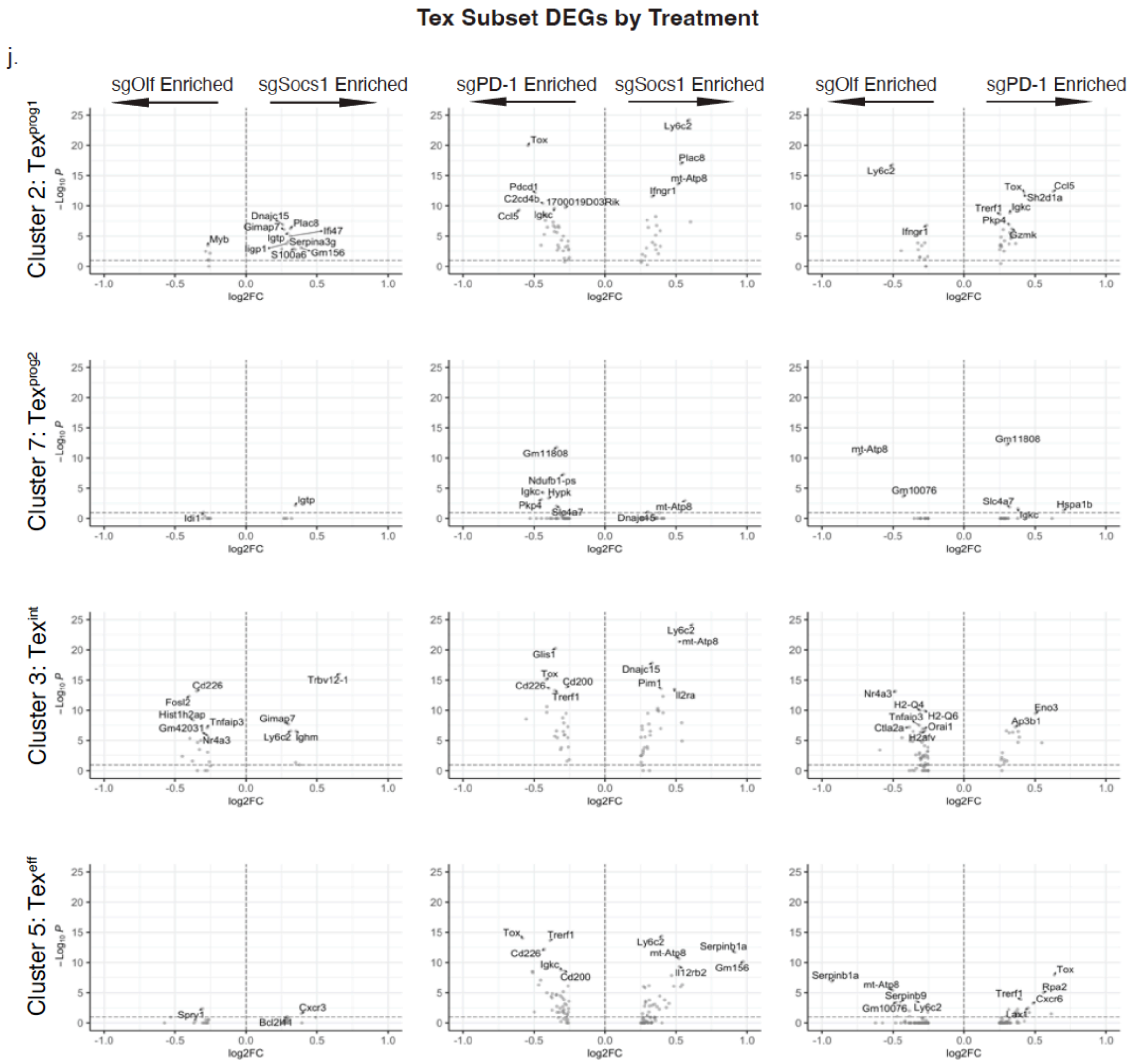

Figure S4:

scRNA-Seq of sgOlf, sgSocs1 and sgPD-1 OT1s from the TME 7 days following transfer. (a) Distribution of general lymphocytes populations from CD45+ cells from the TME. Cells were annotated computationally, with the Haemosphere mouse RNA-Seq database(3) used to verify the predominate lymphocyte clusters. (b) General lymphocyte population frequency per treatment group. **(c)** Cluster-wise cell counts per treatment group. **(d)** Top 10 DEGs (by avgLog2FC) per cluster. Significance was determined using the Wilcoxon Rank Sum test and genes with an adjusted p value < 0.1 were considered significant. **(e)** UpSet plot of DEG counts between treatment groups within the OT1 population. **(f)** Heatmap of median GSVA scores of Miller et al.(3) Tex gene signatures and Beltra et al.(4) Tex gene signatures per Tex cluster by treatment group. **(g)** Boxplot of GSVA scores of five TCR activation-related gene signatures from Reactome by Tex cluster. **(h)** Barplot of T cell cluster frequency by treatment group. **(i)** UpSet plots of DEG counts between treatment groups per Tex cluster. **(j)** Volcano plots depicting DEGs

between treatment groups per Tex cluster within the OT1 population. Names of the top 10 genes (by adjusted p value) are highlighted for each comparison. If fewer than 10 genes have an adjusted p value < 0.1 for a comparison, only the names of genes with an adjusted p value < 0.1 are highlighted.

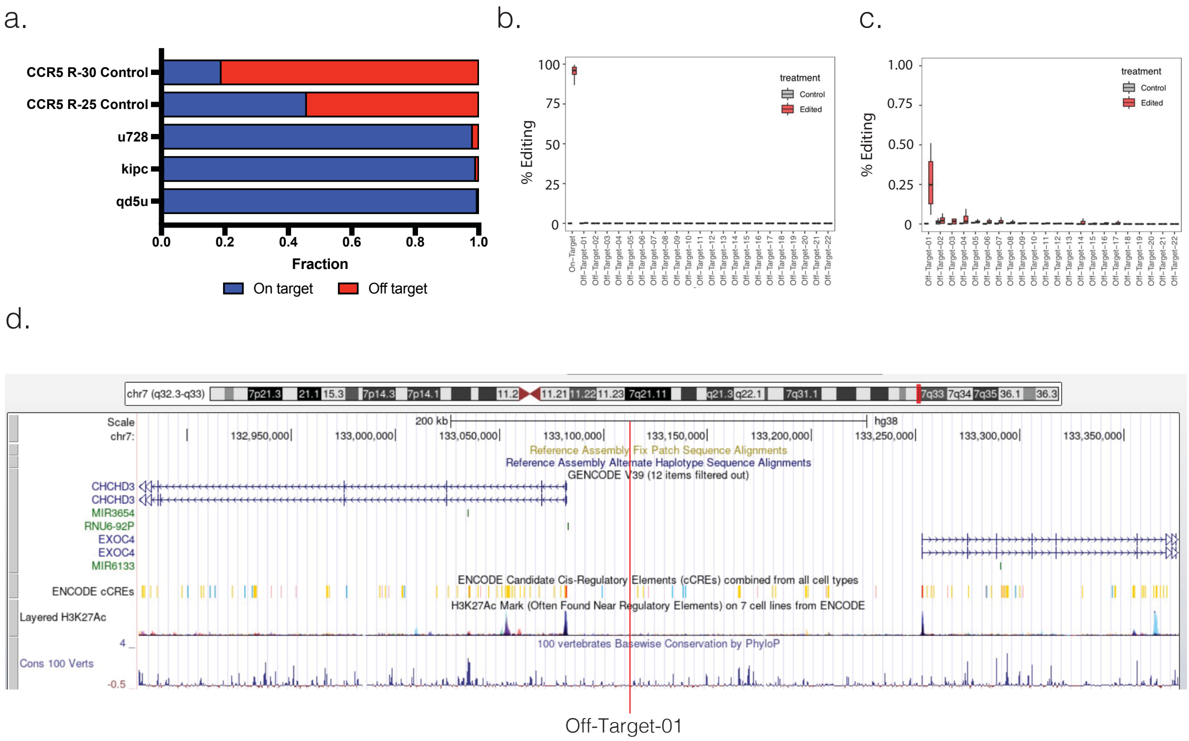

Figure S5: Identification of the genomic locations of u728 sgRNA off-target cut sites
(a) GUIDE-Seq was used to quantify and characterize the frequency of on- and off-target cut sites in primary human T cells. Two sgRNAs targeting CCR5 (R-30 and R-25) with high off-target editing frequencies were included as benchmarks, with the SOCS1 on-target editing efficiency depicted in blue, and the cumulative editing efficiency of off-target loci depicted in red for the u728, kipc and qd5u sgRNAs. **(b)** Amp-Seq confirmation of on- and off-target editing for the u728 sgRNA in human TIL including 22 potential off-target sites identified in (a). The u728 sgRNA achieved a median of 96% editing of the SOCS1 gene. Off-Target-01 confirmed as reaching statistically significant levels of 0.3%. **(d)** The genomic location of the Off-Target-01 site is in an intragenic region on Chromosome 7 with no known regulatory regions.

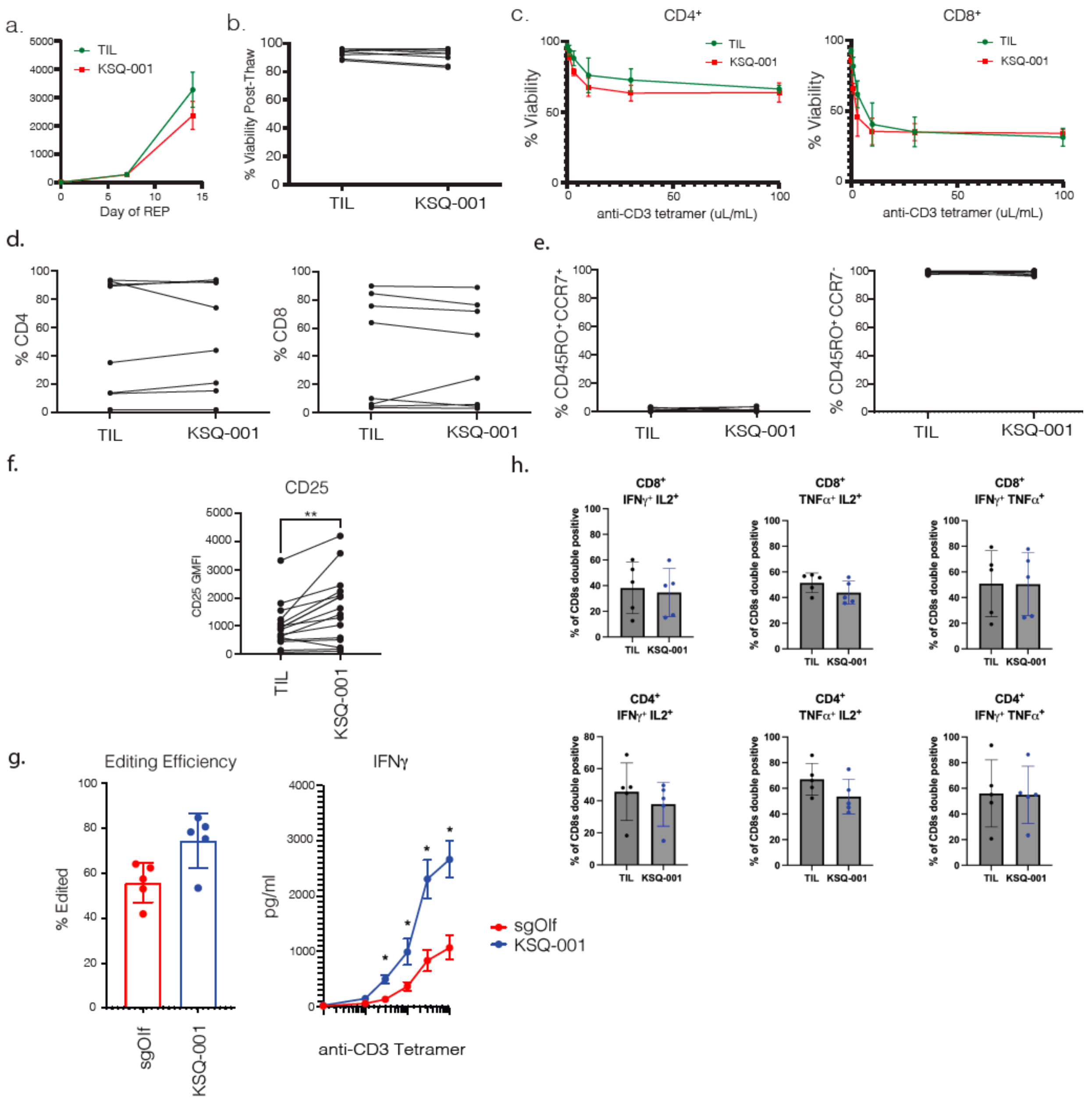

Figure S6: Characterization of KSQ-001

TIL and KSQ-001 were manufactured. (**a**) Fold expansion of TIL and KSQ-001 cells over 14 days of REP. (**b**) Following manufacture and cryopreservation, TIL and KSQ-001 were thawed and viability assessed by AOPI staining, with each dot reflecting an independent paired donor (**c**) To evaluate the ability of KSQ-001 to undergo activation-induced cell death (AICD) following activation, cryopreserved TIL and KSQ-001 cells were thawed, rested overnight, and activated with the indicated concentration of CD3 tetramer for 18 hours. The AICD profile of CD4 and CD8 within TIL and KSQ-001 was measured by FACS analysis of Caspase-3 staining. (**d**) The frequency of donor-paired CD4 and CD8 cells within KSQ-001 and TIL is depicted. Each dot reflects an independent donor. (**e**) The frequency of central memory cells (CCR7^{+}, CD45RO^{+}) and effector

memory cells ($CCR7^-$, $CD45RO^+$) in TIL and KSQ-001 following manufacture are displayed. **(f)** CD25 expression of TIL and KSQ-001 across independent donors. **(g)** TIL and KSQ-001 were manufactured with the same donor, with TIL undergoing electroporation of sgRNA/Cas9 RNPs targeting an Olfactory gene (sgOlf). Editing efficiency of the Olfactory and SOCS1 genes are depicted in the graph on left, and IFNg production following activation with anti-CD3 tetramers at the indicated concentration is shown on the right. **(g)** Polyfunctionality of TIL and KSQ-001 from five independent donors was assessed, with frequency of CD8 or CD4 T cells producing multiple cytokines by intracellular cytokine stain (ICS) assessed as indicated. Each dot reflects an independent donor, with ** = p value <0.01 and * = < 0.05 by a Student's *t* test between the indicated comparator groups.

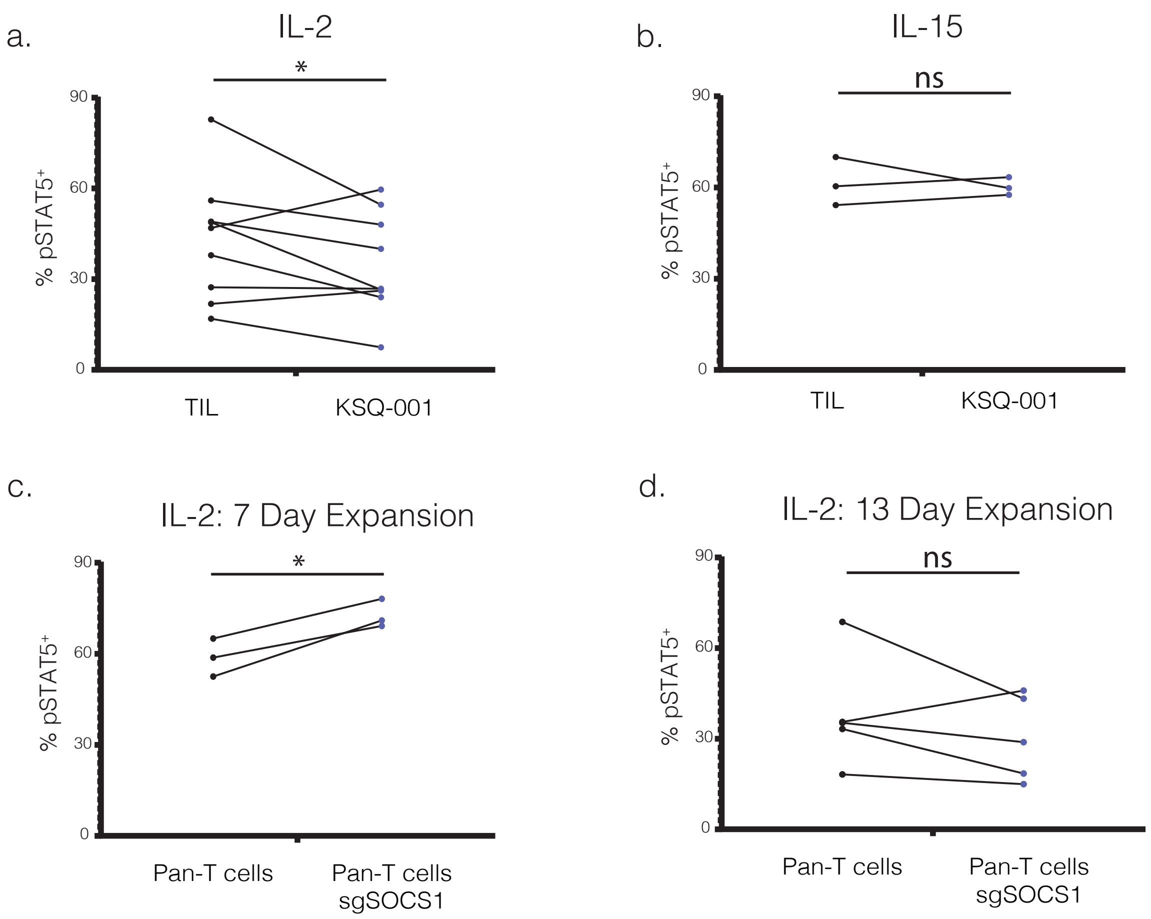

Figure S7: Sensitivity of KSQ-001, TIL, and sgSOCS1-edited $CD3^+$ T cells to IL-2 and IL-15
The sensitivity of KSQ-001 and TIL to IL-2 and IL-15 through STAT5 phosphorylation (pSTAT5) was evaluated. Following REP and cryopreservation, donor-paired TIL and KSQ-001 were thawed, rested, and activated with the indicated cytokine. **(a)** pSTAT5 signals between TIL and KSQ-001 following IL-2 activation. **(b)** pSTAT5 signals between TIL and KSQ-001 following IL-15 activation. **(c)** $CD3^+$ Pan-T cells were activated and edited sgSOCS1, with sensitivity to IL-2 through pSTAT signals evaluated at 7 days following activation in comparison to unengineered cells. **(d)** Same experiment as in (c), but with pSTAT5 sensitivity to IL-2 evaluated at 13 days following initial activation. Statistical significance: * = $p < 0.05$ using a Student's *t* test between the indicated groups. ns = not significant

Supplementary References

1. Tsai SQ et al. GUIDE-Seq enables genome-wide profiling of off-target cleavage by CRISPR-Cas nucleases. *Nat Biotechnol* 2015;33(2):187–197.

2. Chen B-M et al. The Affinity of Elongated Membrane-Tethered Ligands Determines Potency of T Cell Receptor Triggering. *Front Immunol* 2017;8:793.

3. Miller BC et al. Subsets of exhausted CD8+ T cells differentially mediate tumor control and respond to checkpoint blockade. *Nat Immunol* 2019;20(3):326–336.

4. Beltra J-C et al. Developmental Relationships of Four Exhausted CD8+ T Cell Subsets Reveals Underlying Transcriptional and Epigenetic Landscape Control Mechanisms. *Immunity* 2020;52(5):825-841.e8.
